# Towards meaningful engagement: theoretical approaches guiding the participation of people living with multiple long-term conditions in health research – a scoping review

**DOI:** 10.1136/bmjph-2025-004602

**Published:** 2026-09-25

**Authors:** Mansi Chopra, Priyobrat Rajkhowa, Shishirendu Ghosal, Deborah Ikhile, Niharika Rao, Mansi Gupta, Shabana Cassambai, Nikhil Srinivasapura Venkateshmurthy, Natalia Oli, Ambika T Pachya, Patrick Highton, Justin Waring, Sailesh Mohan, Abhinav Vaidya, Kuldeep Singh, Kamlesh Khunti, Dorairaj Prabhakaran, Monika Arora

**Affiliations:** 1 HRIDAY, New Delhi, India; 2 University of Leicester, Leicester, UK; 3 Centre for Chronic Disease Control, New Delhi, India; 4 Kathmandu Medical College, Kathmandu, Nepal; 5 Department of Community Medicine, Kathmandu Medical College, Kathmandu, Nepal; 6 Social Sciences and Humanities, Loughborough University, Loughborough, UK; 7 Department of Pediatrics, All India Institute of Medical Sciences, Jodhpur, Rajasthan, India

**Keywords:** Public Health, Community Health, Community Participation

## Abstract

The rising prevalence of multiple long-term conditions (MLTCs), largely driven by non-communicable diseases (NCDs), presents complex healthcare challenges. Evidence suggests that meaningful engagement of people with lived experience can improve intervention relevance, health outcomes and equity. Theories, frameworks and models (TFMs) may provide structured approaches to support such engagement among people living with MLTC (PLwMLTC).

**Objectives:**

To identify TFMs informing engagement strategies for PLwMLTC.

**Design:**

Scoping review followed the Arksey and O’Malley and Levac *et al* methodological frameworks and reported according to the Preferred Reporting Items for Systematic Reviews and Meta-Analyses Extension for Scoping Reviews guidelines.

**Data sources:**

PubMed, Scopus, CINAHL and Embase databases were searched on 20 December 2024 without date restrictions.

**Eligibility criteria:**

Studies describing TFMs adopted to support engagement and involvement of PLwMLTC and community members.

**Data extraction and synthesis:**

Data were extracted using a predefined charting form and synthesised narratively.

**Results:**

Of 756 articles screened, 25 met inclusion criteria. Commonly adopted TFMs included the Chronic Care Model, Collaborative Care Model, Health Belief Model, Transtheoretical Model, Patient-Centred Medical Home, Improving Mood–Promoting Access to Care Model and Patient-Centred Care Model. These TFMs facilitated meaningful engagement through patient education, behaviour change programmes, community-based care, peer support and coordinated provider–patient interactions. Evidence linked several TFMs to improvements in clinical outcomes (eg, blood pressure and depression), while others primarily influenced sociobehavioural dimensions. Persistent challenges concerned heterogeneity in integrating engagement within health systems and limited evidence on contextual adaptation across diverse settings, particularly in relation to NCD-driven MLTC.

**Conclusions:**

TFMs provide structured approaches to engage PLwMLTC meaningfully, through behaviour change strategies, multidisciplinary care coordination and community network strengthening. Effective implementation relies on ensuring cultural relevance and alignment with local health system capacities. Future research should focus on adaptation, implementation, sustainability, scalability and equity across diverse settings.

**WHAT IS ALREADY KNOWN ON THIS TOPIC?**

**WHAT THIS STUDY ADDS?**

- This scoping review examines and synthesises the range of theories, frameworks and models (TFMs) employed to guide the engagement of people living with MLTC globally. It elucidates their operational mechanisms, associated clinical and sociobehavioural outcomes and identifies persisting gaps in integration and contextual adaptation within health systems, particularly across heterogeneous social, cultural and economic settings. The review not only delineates the effectiveness and limitations of existing TFMs but also charts pathways for advancing more inclusive, participatory and community-grounded engagement approaches in MLTC research and practice.

**HOW THIS STUDY MIGHT AFFECT RESEARCH, PRACTICE OR POLICY?**

- The insights derived from this review offer practical and research-oriented guidance for the selection, adaptation, and contextualization of TFMs to strengthen engagement strategies in MLTC and NCDs. They underscore the importance of cultural appropriateness, meaningful community engagement, and health system responsiveness in advancing equity and scalability. The findings have direct implications for researchers, practitioners, and policymakers in co-designing people-centred, integrated strategies, and in shaping participatory implementation research and programmes aimed at improving outcomes for individuals and populations living with MLTC on a global scale.

## Introduction

 Multiple long-term conditions (MLTC), defined as the coexistence of two or more chronic health conditions within an individual, is rising globally with an estimated pooled prevalence of 37.2% (95% CI 34.9% to 39.4%).^1,2^ This increases significantly among those over the age of 60 years.^2^ A substantial proportion of MLTC is driven by non-communicable diseases (NCDs), including diabetes, cardiovascular diseases, cancers and mental health conditions, which collectively account for the major share of the global chronic disease burden. This poses a significant challenge to healthcare systems, given the complex interplay of physical, mental, social and economic factors affecting those living with MLTC.^3^ Traditional healthcare approaches often fail to address these multifaceted needs of patients, emphasising the need for a shift towards person-centred and community-driven strategies.^3^ Despite the growing burden, research on MLTC remains limited.^4^ The complex challenges of MLTC necessitate cocreated solutions with beneficiaries and communities, grounded in a deep understanding of their needs and contextualised integration of strategies to achieve broader and sustainable health outcomes.

Evidence from studies and the WHO highlights the transformative potential of meaningful patient and public involvement and engagement in healthcare research.^5,6^ Meaningful engagement refers to the respectful, equitable and dignified inclusion of individuals with lived experience in decision-making, research and healthcare processes within enabling environments that recognise lived experience as a form of expertise and support shared influence over decisions affecting health and well-being.^7^ Meaningful involvement of people living with long term conditions such as cancer, mental health, AIDS among others, has been shown to improve physical health outcomes, enhance mental well-being and empower individuals to take greater control over their health.^8–10^ Beyond individual benefits, these engagements foster stronger community networks, reduce healthcare disparities and promote social inclusion.^11^ Such benefits through meaningful engagement for health promotion are not merely confined to the health domain but extend to social and economic upliftment, ultimately improving the quality of life for both individuals and their communities.^12^ However, the complexity of MLTC, particularly those associated with multimorbidity of NCDs, presents unique challenges that necessitate tailored engagement strategies, emphasising the urgency of systematically exploring this area.^13^

Theory/frameworks/models (TFMs) provide structured approaches to engagement and are integral to ensuring effectiveness in managing and improving health. Widely adopted models, such as the Patient-Centred Care Model (PCCM) and community-based participatory research (CBPR), have demonstrated their value in aligning research with the needs and values of individuals and communities.^14,15^ Similarly, TFMs like the Collaborative Care Model (CoCM) have been pivotal in promoting health awareness, behaviours and motivations for reducing depression and improving self-management of chronic disease.^16^ Despite their success in other health domains, the application of these approaches to meaningfully engage people living with MLTC (PLwMLTC) and their communities, especially those affected by NCD-driven MLTC, remains underexplored. Moreover, the lack of a comprehensive understanding of the strengths and limitations of these TFMs hinders the development of contextually relevant and effective strategies for this population.

In this context, as part of the National Institute for Health and Care Research (NIHR) Global Health Research Centre (GHRC) for MLTC research, this scoping review is situated within the formative phase of an extensive Community Engagement and Involvement (CEI)-driven research programme aimed at codesigning a comprehensive, integrated community engagement and person-centred intervention model. This process is built on participatory, dialogue-based CEI activities, such as community conversations, forming community advisory boards, identifying and mobilising local champions and using culturally rooted communication methods like nukkad nataks (street plays). Additionally, two structured CEI-based educational courses were developed, one tailored for healthcare professionals and another for community members including PLwMLTC and caregivers, following iterative inputs gathered during the conversations and interviews. These initiatives were conceptualised to institutionalise meaningful engagement practices and build long-term capacities among both system actors and communities for sustained cocreation of solutions to address MLTC, particularly in the context of NCDs.

Thus, this scoping review seeks to identify and map the widely used TFMs to engage and involve PLwMLTC and community members globally. Through an examination of their conceptual foundations, implementation approaches and reported engagement mechanisms, the review aims to clarify how engagement is structured and operationalised within different TFMs across contexts, without assessing comparative effectiveness, care delivery performance or clinical outcomes. Ultimately, the findings will help shape a healthcare approach that goes beyond clinical management of MLTC, placing equal emphasis on the meaningful engagement and empowerment of individuals and their communities, and ensuring that research and interventions are grounded in and responsive to their lived experiences and priorities.

## Materials and methods

This scoping review was conducted by adopting the frameworks presented in ‘Arksey and O’Malley’ and ‘Levac *et al’*.^17,18^ To ensure adequate coverage of necessary elements in reporting, the ‘Preferred Reporting Items for Systematic Reviews and Meta-Analyses’ (PRISMA) extension for Scoping Reviews, checklist was adopted.^19^ The subsequent sections elaborate on the specific stages employed during this scoping review. Additionally, the review was prospectively registered in the online open-access repository Figshare, with the DOI: 10.6084/m9.figshare.22778333.

### Stage 1: identifying the review question

The review question and the objective were formulated after a thorough literature search and brainstorming:

1. What are the widely used TFMs to engage and involve PLwMLTC and community members globally?

This scoping review adopted the Population, Concept, Context (PCC) format, according to the Joanna Briggs Institute manual for evidence synthesis 2020 for developing the research question, as provided in table 1.^20^

**Table 1:** PCC framework for developing the research question

No.	PCC framework	Description
1	Population (P)	PLwMLTC and community members associated with PLwMLTC. The list of disease/health conditions is included in online supplemental file 1.^57^
2	Concept (C)	TFMs used for engaging and involving the communities or PLwMLTC.
3	Context (C)	Global setting.

PLwMLTC, people living with multiple long-term condition; TFMs, theories, frameworks and models.

### Stage 2: identifying relevant studies

The search strategy for this study involved a comprehensive search across several databases, including Embase, Scopus, Medline (PubMed) and CINAHL, to identify relevant peer-reviewed articles published in English. The search was conducted on 20 December 2024 with no timeline restrictions. Boolean operators (AND, OR) were used to combine key concepts and refine the results. For example, terms such as “community engagement,” “long-term conditions,” “theories,” “models,” “frameworks,” and “multiple long-term conditions” were combined using AND/OR operators to ensure comprehensive retrieval of relevant studies. To specifically capture evidence on MLTC, combinations of individual diseases were also employed (for instance, “diabetes AND hypertension,” “hypertension AND cancer,” or “diabetes AND cardiovascular disease”), thereby reflecting the coexistence of conditions central to MLTC definitions. This dual approach ensured the inclusion of both conceptual literature and disease-specific combinations relevant to MLTC, yielding a total of 982 articles. Furthermore, to ensure comprehensive coverage, the reference lists of the included papers were also reviewed to incorporate all relevant literature. An overview of the electronic search across databases is provided in online supplemental file 2.

### Stage 3: study selection

Retrieved papers’ titles and abstracts underwent screening by two reviewers (PR and SG), assessing eligibility through predefined criteria on Covidence, a systematic review web-based management platform.^21^ In case of uncertainty, assistance from a third researcher (MC) was sought. Full texts of tentatively eligible studies (n=64) were retrieved after the initial screening and independently reviewed by the two reviewers (PR and SG). The included studies (n=25) underwent data charting for the final scoping review, detailed in a flow chart following the^22^ diagram as provided in figure 1.^22^ All studies meeting predefined criteria, irrespective of quality, were included (table 2). This review included studies with quantitative, qualitative and/or mixed research methods.

**Table 2:** Inclusion and exclusion criteria

Criteria	Inclusion	Exclusion
TFMs	Studies discussing widely used TFMs for engaging and involving PLwMLTC and community members in their interventions.	Studies that merely applied/adopted widely recognised TFMs for analytical or interpretative purposes, such as structuring data analysis or informing the discussion, without integrating these frameworks into their interventions.
Study design	Original articles (both qualitative, quantitative and/or mixed-methods studies). Relevant case studies, book chapters and grey literature using any community engagement and involvement framework. Descriptive observational studies (eg, case reports, case series, descriptive cross-sectional studies)	Review articles, conference proceedings, editorial letters, correspondence, abstracts, author debates, summary articles, interviews.
Language	English language articles were included	Non-English language articles
Year	No limitation	No limitation
Geography	Global level	No limitation

PLwMLTC, people living with multiple long-term condition; TFMs, theories, frameworks and models.

**Figure 1:**
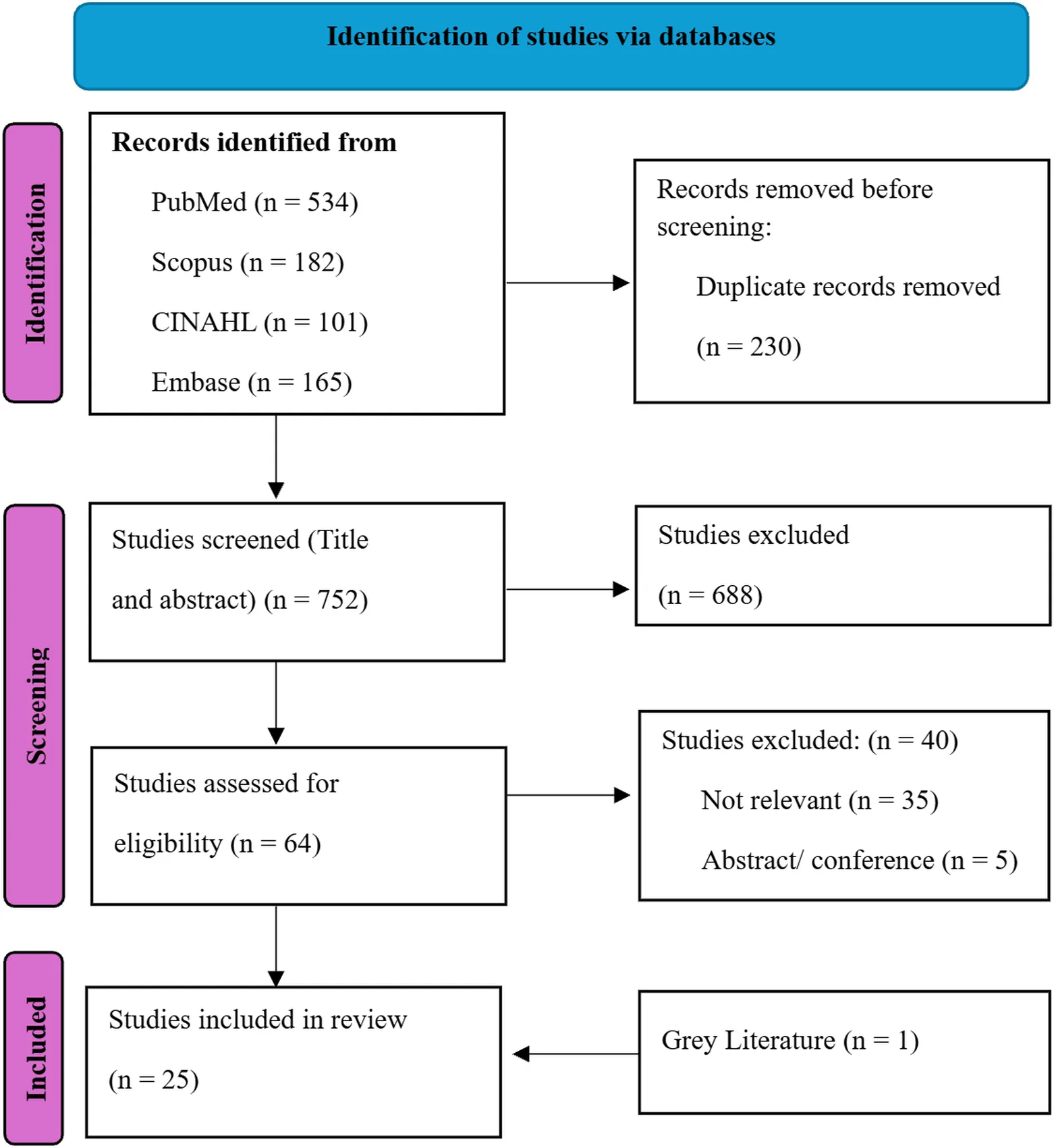
PRISMA flow diagram.

### Stage 4: charting the data

Data charting was performed by adopting a predefined data charting form, provided in online supplemental file 3. Each article included in the review was independently charted by two reviewers (PR and SG), using Microsoft Excel 2505. In cases of disagreement, a third reviewer (MC) was consulted. The charting process covered various aspects such as participants, context, concepts, study methods, theories, models or frameworks used, followed by their additional critical findings relevant to the review question, like positive and negative aspects of implementation. Data extraction from included studies was completed accordingly.

### Stage 5: collating, summarising and reporting the results

The study employed narrative synthesis, using tables to summarise study characteristics and findings. A comprehensive list of countries and sources was compiled to identify relevant literature. Qualitative data from the charting table was used to map and report on theories/models/frameworks to engage and involve PLwMLTC and community members globally. Consistent with scoping review methodology, critical appraisal of included sources was not undertaken, as the objective of the review was to identify, map and synthesise the range of TFMs rather than to assess the methodological quality or effectiveness of individual studies.

## Results

A comprehensive literature search yielded 982 records. After removing 230 duplicates, 752 titles and abstracts were screened based on predefined eligibility criteria (table 2). Of these, 64 articles underwent full-text review, resulting in the inclusion of 24 studies. Additionally, one relevant source identified through a targeted Google search was classified as grey literature, bringing the final number of included studies to 25 (figure 1).

Majority of the studies (16 out of 25; 60%) were conducted in high-income countries (HICs)—USA (n=13),^23–35^ Australia (n=2, 8%)^36,37^ and the UK (n=1, 4%).^16^ Among the rest, five studies, 20% were from India,^38–42^ two each, 8% from China^43,44^ and Africa, respectively. One conducted in Malawi,^45^ and one jointly conducted in Kenya and Uganda (online supplemental file 4).^46^

Most of the study methods used were experimental studies: 10 (40%) randomised controlled trials (RCTs),^16,25–27,31,38,39,42–44^ 2 (8%) pre–post design,^33,46^ 3 (12%) quasi-experimental studies,^34–36^ 5 (20%) secondary data analysis of previously conducted RCTs,^29,30,40,41,46^ followed by 3 qualitative (12%),^23,28,45^ and the remaining 2 studies (8%), 1 each were cross-sectional design^32^ and longitudinal observational design.^24^

### Elements across TFMs supporting meaningful engagement of PLwMLTC

A diverse set of TFMs were identified across the included studies, each providing structured approaches to facilitate meaningful engagement of PLwMLTC. These TFMs guided health education, patient activation, collaborative care, and behavioural interventions while providing insights into system-level feasibility. Importantly, clinical and behavioural outcomes were variably reported, offering illustrative evidence of how engagement strategies linked to these TFMs translated into health benefits. Collectively, these findings demonstrate that structured, theory-driven approaches are critical for both sustaining patient participation and improving health-related outcomes in PLwMLTC.

A wide range of chronic conditions were reported in the included studies. Hypertension and stroke were examined in multiple studies (n=3),^25,43,44^ while hypertension and diabetes were also frequently investigated (n=2).^33,46^ Several studies (n=11) focused on mental health comorbidities with chronic conditions, including depression, diabetes, and cardiovascular disease,^16,23^ depression and diabetes alone,^31,38,41^ and depression, diabetes and ischaemic or coronary heart disease.^27,39,42^ Depression combined with anxiety-related disorders such as post-traumatic stress disorder (PTSD), panic disorder, and generalised anxiety disorder was also explored.^30,32,40^ Other studies (n=5) highlighted HIV in relation to depression,^28^ haemophilia,^24,35^ hypertension,^45^ and alcohol use disorder.^26^ Broader MLTC patterns (n=3) were observed in studies covering congestive heart failure, diabetes, hyperlipidaemia, asthma, COPD,^34^ as well as chronic obstructive pulmonary diseases^36^ and chronic diseases in general.^37^ Depression with associated comorbidities (n=1) was also noted.^29^

### Collaborative care model

The CoCM was among the most widely adopted TFMs for PLwMLTC, particularly to integrate mental healthcare with the management of physical chronic conditions. Meaningful engagement was facilitated through structured health education, behavioural counselling and self-management training, enabling patients to actively participate in their own care. Engagement extended to caregivers, who were involved in collaborative goal-setting, family support and participation in community-based or digital support groups. Multidisciplinary teams, including primary care providers, mental health specialists, care coordinators and social workers, ensured coordinated delivery across rural health centres, HIV programmes, diabetes clinics and haemophilia care services.^16,23,27,28,30–32,34,35,38–42^

Clinical outcomes demonstrated reductions in depression, low-density lipoprotein (LDL) cholesterol and Systolic blood pressure (SBP), alongside decreased hospitalisations, indicating that structured engagement translated into measurable health benefits. Sociobehavioural outcomes included enhanced coping strategies, increased participation in psychotherapy and higher patient-centred care ratings. At the system level, CoCM was feasible for embedding non-physician care coordinators and leveraging digital platforms to support sustained engagement. Together, these findings suggest that CoCM was commonly used to support meaningful engagement among PLwMLTC and was associated with reported clinical and psychosocial improvements across diverse study settings, although findings should be interpreted in light of variations in study designs, populations and contexts.

### Chronic care model

The chronic care model (CCM) was frequently adopted to guide structured, proactive care for PLwMLTC. Meaningful engagement was supported through tailored health education, counselling, digital coaching and family-centred interventions. Patients and caregivers actively participated in collaborative goal-setting, routine follow-ups and group-based community activities, coordinated by multidisciplinary teams including physicians, nurses, psychiatrists, therapists and social workers. Shared responsibility between patients and providers fostered sustained participation and empowered self-management.^25,33^

Clinical outcomes were mixed but informative. While one study found no significant change in SBP, participants in the intervention group achieved a 15% greater reduction in salt intake, illustrating behaviour change despite limited clinical translation. Another study reported reductions in SBP and DBP, particularly among participants with higher engagement. Beyond individual outcomes, CCM enhanced provider adherence to guidelines, patient activation and self-management behaviours. System-level implementation was feasible in implementation settings and suggested potential for cost reduction and improved lived experiences, though low participation and structural barriers limited effectiveness. Overall, CCM illustrates how TFMs can structure meaningful engagement of PLwMLTC, supporting behavioural change and system-level integration.^25,33^

### Health belief model

The Health Belief Model (HBM) was adopted to interventions targeting perceptions of risk, benefits and barriers to care among PLwMLTC. Engagement was operationalised through multimethod health education, including tailored counselling, digital modules, reminders and peer-sharing sessions. Patients participated in interviews, follow-ups and cocreated care plans, with collaborative support from nurses, physicians and counsellors. By centring patient perceptions, HBM fostered dialogue, empowered self-management and promoted active participation in care.^36,44,45^

Clinical outcomes included reductions in SBP, low stroke recurrence and improvements in physical activity, nutrition, medication adherence and QoL. Sociobehavioural gains comprised increased self-efficacy, motivation for lifestyle change and sustained patient activation. HBM interventions were feasible across hospital settings using face-to-face, telephone and SMS-based modalities, although structural barriers such as medication costs highlighted the need to address social determinants alongside engagement. Overall, HBM demonstrates how a theory-driven framework can structure meaningful engagement while delivering health and behavioural benefits care.^36,44,45^

### Improving mood–promoting access to collaborative treatment model

The Improving Mood–Promoting Access to Collaborative Treatment (IMPACT) model targeted depression care for older adults with multimorbidity. Meaningful engagement involved tailored information, structured behavioural activation, shared decision-making and psychotherapy follow-ups. PLwMLTC were supported to articulate treatment goals, with ongoing engagement maintained through regular monitoring and motivational support. Multidisciplinary teams coordinated integrated delivery of psychological and physical interventions.^29^

Clinical outcomes included reductions in depression severity, higher remission rates, improvements in mental health scores and enhanced QoL. Sociobehavioural analyses indicated that patients with greater comorbidity burden responded as effectively as those with fewer conditions, highlighting the broad applicability of meaningful engagement. At the system level, IMPACT was feasible across primary care and community-based programmes and integrated into routine practice without extensive new infrastructure. Overall, IMPACT provides a framework for meaningful engagement and evidence-based depression management in PLwMLTC.^29^

### Transtheoretical model

Transtheoretical model (TTM) was applied to promote progressive behavioural change among PLwMLTC, particularly for alcohol reduction and safer sex practices in populations with haemophilia and HIV. Engagement was structured around the stage-based approach, supporting patients through precontemplation, contemplation, preparation, action and maintenance. Participation was facilitated through motivational interviewing, peer discussions, social activities and weekend retreats, providing social reinforcement and reflection on readiness to change.^24,26^

Clinical outcomes included 79% of participants achieving and maintaining action/maintenance stages for safer sex, with sustained reductions in alcohol use over 12 months. Sociobehavioural benefits included enhanced self-efficacy, motivation and increased family and peer support. TTM-based programmes were feasible across multiple sites, demonstrating that structured engagement can drive sustained behavioural improvements in socially complex populations.^24,26^

### Patient-centred medical home

Patient-Centred Medical Home (PCMH) enhanced individualised, coordinated care for PLwMLTC in primary care. Engagement involved codesign of care plans, self-management education and digital monitoring tools, with participation supported through shared decision-making and general practitioner-led multidisciplinary teams.^37^ Clinical outcomes demonstrated improvements in patient activation, with higher baseline activation predicting better clinical and behavioural results. Disparities were noted among older patients and those without private insurance. System-level implementation was feasible, scalable and integrated into routine practice. Overall, PCMH illustrates how patient-centred frameworks can facilitate meaningful engagement while delivering measurable health benefits.^37^

### Patient-centred care model

PCCM integrated culturally tailored, patient-centred approaches into HIV and chronic disease management, particularly in sub-Saharan Africa. Engagement included multidisease screening, linkage-to-care activities and patient participation in care planning, supported by multidisciplinary teams embedded in local health systems.^46^

Clinical outcomes included a 21% reduction in all-cause mortality and 22% improvement in hypertension control over 3 years. Higher engagement correlated with improved diagnosis, treatment linkage and adherence. Sociobehavioural outcomes included stronger community trust and participation in screening programmes. System-level feasibility and scalability were demonstrated, especially in resource-constrained rural settings. PCCM shows that culturally tailored frameworks can achieve meaningful engagement of PLwMLTC while improving population health outcomes.^46^ Across several TFMs, engagement outcomes were influenced by broader social and structural factors, including medication affordability, access to care, insurance coverage and resource constraints. For example, HBM-based interventions identified medication costs as barriers to engagement, while PCMH studies highlighted disparities associated with insurance status, and PCCM demonstrated implementation within resource-constrained settings. Although social determinants of health were not the primary focus of the included TFMs, these findings suggest that meaningful engagement may be enhanced when implementation strategies are attentive to the wider social and structural factors shaping participation, access and self-management among PLwMLTC.

## Discussion

This study identified various TFMs employed to meaningfully engage and involve PLwMLTC in health research. Each identified TFM demonstrated distinct strengths and limitations, particularly in relation to enhancing self-management, strengthening patient–provider communication, fostering active participation in health research, and informing health promotion strategies.

MLTC present a complex challenge that requires integrated, person-centred care strategies. PLwMLTC often experience fragmented care, polypharmacy, and increased risk of poor health outcomes.^47^ Meaningful engagement of PLwMLTC, guided by TFMs, can help address these complex challenges through health education and collaborative care, while also strengthening CEI in health research. Among the TFMs examined, the CoCM was the most frequently reported framework and was associated with improvements in depression and other chronic disease-related outcomes across several included studies.^16,23^ These findings are consistent with previous literature reporting positive outcomes associated with CoCM implementation in MLTC care.^38^ Although several included studies reported positive individual-level outcomes associated with CoCM implementation, important questions remain regarding its dissemination, contextual adaptation, and implementation across different health systems. Achieving successful dissemination will necessitate overcoming organisational-level barriers, addressing challenges in clinical practice, and securing creative financing to support the implementation of team-based care.^48^ These observations highlight the need for further research to understand the conditions under which CoCM may support meaningful engagement among PLwMLTC across diverse settings. A more concerted effort is needed to better understand how collaborative care approaches can be adapted and implemented across varying contexts while maintaining meaningful engagement of PLwMLTC.

The CCM emerged as another TFM but yielded mixed results. Although the CCM has been widely acknowledged for its effectiveness in chronic disease management, several studies in this review found only limited improvements in MLTC outcomes compared with standard care.^49^ This contrasts with earlier findings that highlight CCM’s strength in improving healthcare delivery.^49^ The mixed outcomes observed in this study may reflect variations in intervention fidelity, resource constraints or population-specific factors. Greater emphasis on capacity building, staff training, and tailored implementation strategies may enhance CCM’s effectiveness in improving healthcare delivery for MLTC through meaningful engagement and involvement of the PLwMLTC. Additionally, embedding CCM within broader health promotion frameworks that foster multidisciplinary collaboration may enhance its effectiveness in addressing complex care needs.

The PCMH model emphasises meaningful engagement and involvement of PLwMLTC through continuous, coordinated and person-centred care, making it particularly relevant for PLwMLTC. Unlike behaviour-centric approaches such as the HBM, the PCMH fosters engagement through team-based care and shared decision-making.^50,51^ Evidence from studies demonstrated that PCMH implementation led to improved patient outcomes in long-term conditions.^51^ While the PCMH model seeks to deliver holistic, person-centred care, its focus on clinical settings can limit the integration of community-based participatory approaches. This is a significant gap, as such approaches are often essential for addressing the complex needs of marginalised populations.^51^ This may result in missed opportunities to fully engage with the broader determinants of health that lie beyond the clinical encounter, such as housing, education, transport and food security.

In contrast, place-based models or asset-based community development approaches are often grounded in community settings and prioritise the cocreation of solutions with local stakeholders.^51^ These models more explicitly centre community voices and lived experiences. The PCMH, by comparison, may lack formal mechanisms for the coproduction of care, a core principle in participatory models such as CBPR.^50,51^ For the PCMH to more fully support meaningful, equitable and inclusive care, especially for PLwMLTC, adaptations could include equity-focused design, culturally tailored outreach, and meaningful roles for community advisory boards or advisory councils.^52–54^ Embedding patient governance structures may also help ensure that the PCMH evolves beyond the clinic into a model that genuinely reflects the needs and priorities of diverse populations.^55^

Building on this, meaningful engagement and involvement of PLwMLTC through PCMH demonstrated its potential as a comprehensive and adaptable framework for enhancing patient activation and self-management in MLTC.^34,37^ The meaningful engagement of PLwMLTC through its emphasis on multidisciplinary teams, individualised care planning, and digital integration, the PCMH model addressed core challenges such as fragmented service delivery and low patient engagement, issues particularly relevant for PLwMLTC.^37^ Improvements in PAM scores highlighted its capacity to empower individuals and foster more proactive involvement in managing their conditions.^37^ However, while this framework has shown significant clinical benefits and cost effectiveness, concerns remain regarding its broader applicability. Comparative study emphasises that the success of such TFMs often hinges on contextual enablers like workforce capacity and institutional support, factors that may be lacking in low-resource or underserved settings.^56^ Therefore, to scale and adopt meaningfully the PCMH models, there is a pressing need for rigorous, equity-driven implementation trials that incorporate community-based mechanisms, contextual adaptation and sustained policy support. This approach will be vital for ensuring that such frameworks meaningfully contribute to improved outcomes for PLwMLTC across diverse healthcare landscapes.

The TTM was adopted in multiple studies to promote sustained behaviour change among PLwMLTC, notably in HIV with haemophilia.^24,26^ These interventions tailored strategies for meaningful engagement and involvement led to the participant’s stage of change, enhancing self-efficacy and decisional balance through stage-matched counselling and skills training. Findings revealed measurable progression through the stages from precontemplation to maintenance especially in reducing high-risk behaviours.^35^ Such evidence supports TTM’s adaptability in chronic illness contexts, including MLTC, where behaviour modification is pivotal. Unlike HBM, TTM offers a longitudinal perspective, enabling personalised interventions over time. Integrating TTM into MLTC frameworks may thus enhance patient engagement and promote sustainable self-management.^24,35^

The synthesis of existing models reports that no single TFM is sufficient in isolation to address the complexity of engaging PLwMLTC. Meaningful engagement requires a strategic integration of behavioural, institutional, community and system level and policy approaches. The TFMs identified differed in the level at which engagement was operationalised. Behavioural models primarily targeted individual motivation and self-management, whereas service-delivery and person-centred models emphasised care coordination, shared decision-making and multidisciplinary collaboration. These differences may influence their applicability and effectiveness across diverse populations and health system contexts. Behavioural models such as the HBM and TTM may offer useful insights into individual motivation and readiness for change, while structural models such as CoCM, PCMH and CCM provide mechanisms that may support care coordination and patient-centred care. Simultaneously, structural models like the CoCM, PCMH and CCM provide the foundational mechanisms to enhance care coordination, patient-centredness and quality improvement across settings. These frameworks emphasise the importance of personalised, comprehensive and equity-driven interventions. Yet, the variability in healthcare infrastructure, sociocultural determinants, policy environments and population needs across regions and globally underscores the necessity for flexible, context-specific implementation strategies.

A majority of the included studies (16 out of 25; 60%) originated from HICs, predominantly the USA. This geographic distribution suggests that certain TFMs, such as CoCM and TTM, are more frequently developed, evaluated and implemented within HIC contexts. In contrast, fewer studies from LMICs such as India, China and countries in Africa indicate limited formal adoption, highlighting the need for culturally and contextually adapted engagement strategies for PLwMLTC in these settings.

Finally, the findings from this review have informed the development of a CEI strategy and intervention model for the NIHR GHRC for MLTC research. This strategy incorporates education, behaviour change, peer support, empowerment and strengthening of community networks to enhance meaningful engagement. Future research should prioritise validating adaptable frameworks for diverse contexts, exploring combinations of TFMs and applying implementation science, participatory methods and realist evaluations to understand contextual enablers and barriers. Codesign with people with lived experiences and community stakeholders will remain central to ensuring that engagement strategies for PLwMLTC are relevant, acceptable, sustainable and scalable.

## Conclusions

In conclusion, this scoping review highlights that a diverse range of TFMs, spanning behavioural, structural and person-centred approaches, have been employed globally to meaningfully engage PLwMLTC and community members. TFMs such as CoCM and CCM were commonly used to support collaborative care approaches, while behaviour-focused frameworks such as HBM and TTM were applied to promote lived experience activation and self-management. Across the included studies, meaningful engagement was frequently supported through CEI-related strategies, including health education, participation in decision-making and multidisciplinary collaboration. The predominance of studies from HICs signals a pressing need for greater adaptation in LMICs, where contextual and structural barriers differ. The TFMs identified in this review varied across populations, conditions and health system contexts, suggesting that the selection and application of TFMs should be guided by specific engagement objectives, population needs and contextual requirements

### Strengths and limitations

A key strength of this review is its comprehensive mapping of TFMs used to support meaningful engagement of PLwMLTC across diverse health conditions, populations and settings. By synthesising evidence from a wide range of study designs and geographical contexts, the review provides a broad overview of how engagement has been conceptualised and operationalised within existing TFMs. Furthermore, the review moves beyond simply identifying frameworks by examining their implementation approaches, engagement mechanisms and CEI approaches, thereby generating insights that may inform future research, policy and practice.

However, several limitations should be considered when interpreting the findings. First, the included studies were heterogeneous in terms of populations, health conditions, TFMs, outcomes and implementation settings, limiting direct comparisons across frameworks. Second, most studies originated from HICs, particularly the USA, which may limit the transferability of findings to LMIC settings where health system structures and community engagement mechanisms differ. Third, the review was restricted to English-language publications, which may have resulted in the exclusion of relevant evidence from non-English-speaking contexts. Finally, several TFMs were represented by only one or two studies, limiting the extent to which conclusions can be drawn regarding their broader applicability across different MLTC populations and settings.

## Supplementary material

10.1136/bmjph-2025-004602 online supplemental file 1

10.1136/bmjph-2025-004602 online supplemental file 2

10.1136/bmjph-2025-004602 online supplemental file 3

10.1136/bmjph-2025-004602 online supplemental file 4

## Data Availability

No data are available.
