# Supplementary material for: Towards meaningful engagement: theoretical approaches guiding the participation of people living with multiple long-term conditions in health research – a scoping review
Source: BMJ Public Health. 2026 Sep 25;4(3):e004602. doi: 10.1136/bmjph-2025-004602 (PMC13630058; doi:10.1136/bmjph-2025-004602)
Supplement: Supplementary file 1 [file bmjph-4-3-s001.docx]

**Supplementary File 1**

The list of MLTCs conditions is included in the study

| **Body system** | **Eligible conditions** |
| --- | --- |
| Cardiovascular disease | Stroke, coronary artery disease, heart failure, peripheral artery disease, heart valve disorders, arrhythmia, venous thromboembolic disease, aneurysm, hypertension (treated and untreated) |
| Metabolic and endocrine disease | Diabetes, Addison’s disease, cystic fibrosis, thyroid disorders |
| Respiratory disease | Chronic obstructive pulmonary disease, asthma, COPD, bronchiectasis |
| Neurological disease | Parkinson’s disease, epilepsy, multiple sclerosis, paralysis, transient ischemic attack, peripheral neuropathy, chronic  primary pain |
| Cancer | Solid organ cancers, haematological cancers, metastatic cancers, melanoma, benign cerebral tumours that can cause disability |
| Mental and behavioural disorder | Dementia, schizophrenia, depression, anxiety, bipolar disorder, drug or alcohol misuse, eating disorder, autism, post-traumatic stress disorder |
| Musculoskeletal disease | Connective tissue disease, arthritis, osteoarthritis, long term musculoskeletal problems due to injury, osteoporosis, gout |
| Digestive disease | Chronic liver disease, inflammatory bowel disease, chronic pancreatic disease, peptic ulcer |
| Urogenital disorder | Chronic kidney disease, end stage kidney disease, endometriosis, chronic urinary tract infection |
| Haematological disorder | Anaemia (including pernicious anaemia, sickle cell anaemia) |
| Eye disease | Cataract, vision impairment that cannot be corrected |
| Ear disease | Hearing impairment that cannot be corrected, Meniere’s disease |
| Infectious disease | HIV/AIDS, tuberculosis, chronic Lyme disease, tuberculosis, post-acute covid-19 |
| Congenital disease | Congenital disease and chromosomal abnormalities |
