# Supplementary material for: Towards meaningful engagement: theoretical approaches guiding the participation of people living with multiple long-term conditions in health research – a scoping review
Source: BMJ Public Health. 2026 Sep 25;4(3):e004602. doi: 10.1136/bmjph-2025-004602 (PMC13630058; doi:10.1136/bmjph-2025-004602)
Supplement: Supplementary file 2 [file bmjph-4-3-s002.docx]

**Supplementary File 2**

**Search strategy**

**PubMed 20/12/20204**

**#1 People living with multiple long-term conditions 2,345,894**

"multiple comorbidity"[Title/Abstract] OR "multimorb*"[Title/Abstract] OR "multimorbid*"[Title/Abstract] OR "multimorbidit*"[Title/Abstract] OR "multi morbidit*"[Title/Abstract] OR "multi morbidit*"[Title/Abstract] OR "multi disease*"[Title/Abstract] OR "multi morbid*"[Title/Abstract] OR "multidisease"[Title/Abstract] OR "multiple diseases"[Title/Abstract] OR "multi-disease"[Title/Abstract] OR "multicondition*"[Title/Abstract] OR "multi condition*"[Title/Abstract] OR "multiple condition"[Title/Abstract] OR "multisyndrome*"[Title/Abstract] OR "multi-syndrome"[Title/Abstract] OR "multiple syndrome"[Title/Abstract] OR "multidisorder*"[Title/Abstract] OR "multi-disorder"[Title/Abstract] OR "multiple disorder"[Title/Abstract] OR "multiproblem*"[Title/Abstract] OR "multiple chronic condition*"[Title/Abstract] OR "morbidity burden*"[Title/Abstract] OR "multipatholog*"[Title/Abstract] OR "polypatholog*"[Title/Abstract] OR "comorb*"[Title/Abstract] OR "comorbid*"[Title/Abstract] OR "co morbid*"[Title/Abstract] OR "co morbid*"[Title/Abstract] OR "comorbidit*"[Title/Abstract] OR "co morbidit*"[Title/Abstract] OR "noncommunicable"[Title/Abstract] OR "non communicable"[Title/Abstract] OR "polymorb*"[Title/Abstract] OR "polymorbidit*"[Title/Abstract] OR "poly morbidit*"[Title/Abstract] OR "pluripatholog*"[Title/Abstract] OR "pluri patholog*"[Title/Abstract] OR "Multimorbidity"[MeSH Terms] OR "Multiple Chronic Conditions"[MeSH Terms] OR ((((((((((((("Cardiovascular Diseases"[MeSH Terms] OR "Cardiovascular Disease"[Title/Abstract] OR "disease cardiovascular"[Title/Abstract] OR "Cardiac Events"[Title/Abstract] OR "Cardiac Event"[Title/Abstract] OR "event cardiac"[Title/Abstract] OR "Adverse Cardiac Event"[Title/Abstract] OR "Adverse Cardiac Events"[Title/Abstract] OR "cardiac events adverse"[Title/Abstract] OR "Major Adverse Cardiac Events"[Title/Abstract] OR "Stroke"[MeSH Terms] OR "Stroke"[Title/Abstract] OR "Strokes"[Title/Abstract] OR "Cerebrovascular Accident"[Title/Abstract] OR "Cerebrovascular Accidents"[Title/Abstract] OR "Cerebral Stroke"[Title/Abstract] OR "Cerebral Strokes"[Title/Abstract] OR "stroke cerebral"[Title/Abstract] OR "strokes cerebral"[Title/Abstract] OR "Cerebrovascular Apoplexy"[Title/Abstract] OR "apoplexy cerebrovascular"[Title/Abstract] OR "vascular accident brain"[Title/Abstract] OR "Brain Vascular Accident"[Title/Abstract] OR "Brain Vascular Accidents"[Title/Abstract] OR "Cerebrovascular Stroke"[Title/Abstract] OR "Cerebrovascular Strokes"[Title/Abstract] OR "stroke cerebrovascular"[Title/Abstract] OR "strokes cerebrovascular"[Title/Abstract] OR "Apoplexy"[Title/Abstract] OR "CVA"[Title/Abstract] OR "CVA"[Title/Abstract] OR "stroke acute"[Title/Abstract] OR "Acute Stroke"[Title/Abstract] OR "Acute Strokes"[Title/Abstract] OR "strokes acute"[Title/Abstract] OR "cerebrovascular accident acute"[Title/Abstract] OR "Acute Cerebrovascular Accident"[Title/Abstract] OR "Acute Cerebrovascular Accidents"[Title/Abstract] OR "cerebrovascular accidents acute"[Title/Abstract] OR "Coronary Artery Disease"[MeSH Terms] OR "Coronary Disease"[MeSH Terms] OR "artery disease coronary"[Title/Abstract] OR "artery diseases coronary"[Title/Abstract] OR "Coronary Artery Diseases"[Title/Abstract] OR "Coronary Arteriosclerosis"[Title/Abstract] OR "arteriosclerosis coronary"[Title/Abstract] OR "atherosclerosis coronary"[Title/Abstract] OR "Coronary Atherosclerosis"[Title/Abstract] OR "Left Main Coronary Artery Disease"[Title/Abstract] OR "Left Main Coronary Disease"[Title/Abstract] OR "Left Main Disease"[Title/Abstract] OR "Left Main Diseases"[Title/Abstract] OR "Heart Failure"[MeSH Terms] OR "Cardiac Failure"[Title/Abstract] OR "Heart Decompensation"[Title/Abstract] OR "decompensation heart"[Title/Abstract] OR "Congestive Heart Failure"[Title/Abstract] OR "heart failure congestive"[Title/Abstract] OR "heart failure right sided"[Title/Abstract] OR "heart failure right sided"[Title/Abstract] OR "right sided heart failure"[Title/Abstract] OR "right sided heart failure"[Title/Abstract] OR "heart failure left sided"[Title/Abstract] OR "heart failure left sided"[Title/Abstract] OR "left sided heart failure"[Title/Abstract] OR "left sided heart failure"[Title/Abstract] OR "Myocardial Failure"[Title/Abstract] OR "Peripheral Arterial Disease"[MeSH Terms] OR "arterial disease peripheral"[Title/Abstract] OR "disease peripheral arterial"[Title/Abstract] OR "diseases peripheral arterial"[Title/Abstract] OR "Peripheral Arterial Diseases"[Title/Abstract] OR "Peripheral Artery Disease"[Title/Abstract] OR "artery disease peripheral"[Title/Abstract] OR "artery diseases peripheral"[Title/Abstract] OR "disease peripheral artery"[Title/Abstract] OR "diseases peripheral artery"[Title/Abstract] OR "Peripheral Artery Diseases"[Title/Abstract] OR "Heart Valve Diseases"[MeSH Terms] OR "Heart Valve Disease"[Title/Abstract] OR "valve disease heart"[Title/Abstract] OR "Valvular Heart Diseases"[Title/Abstract] OR "heart disease valvular"[Title/Abstract] OR "Valvular Heart Disease"[Title/Abstract] OR "Heart Valvular Disease"[Title/Abstract] OR "Heart Valvular Diseases"[Title/Abstract] OR "valvular disease heart"[Title/Abstract] OR "arrhythmias, cardiac"[MeSH Terms] OR "arrhythmia cardiac"[Title/Abstract] OR "Arrhythmia"[Title/Abstract] OR "Arrythmia"[Title/Abstract] OR "Cardiac Arrhythmia"[Title/Abstract] OR "Cardiac Arrhythmias"[Title/Abstract] OR "Cardiac Dysrhythmia"[Title/Abstract] OR "dysrhythmia cardiac"[Title/Abstract] OR "Respiratory Sinus Arrhythmia"[MeSH Terms] OR "sinus arrhythmia respiratory"[Title/Abstract] OR "arrhythmia, sinus"[MeSH Terms] OR "arrhythmias sinus"[Title/Abstract] OR "Sinus Arrhythmias"[Title/Abstract] OR "Sinus Arrhythmia"[Title/Abstract] OR "arrhythmias sinoatrial"[Title/Abstract] OR "Sinoatrial Arrhythmias"[Title/Abstract] OR "Sinoatrial Arrhythmia"[Title/Abstract] OR "Thromboembolism"[MeSH Terms] OR "thromboembolism*"[Title/Abstract] OR "Aneurysm"[MeSH Terms] OR "Aneurysms"[Title/Abstract] OR "Fusiform Aneurysm"[Title/Abstract] OR "aneurysm fusiform"[Title/Abstract] OR "aneurysms fusiform"[Title/Abstract] OR "Fusiform Aneurysms"[Title/Abstract] OR "Saccular Aneurysm"[Title/Abstract] OR "Hypertension"[MeSH Terms] OR "blood pressure high"[Title/Abstract] OR "blood pressures high"[Title/Abstract] OR "High Blood Pressure"[Title/Abstract] OR "High Blood Pressures"[Title/Abstract] OR "Diabetes Mellitus"[MeSH Terms] OR "Addison Disease"[MeSH Terms] OR "disease addison"[Title/Abstract] OR "Addison's Disease"[Title/Abstract] OR "Addisons Disease"[Title/Abstract] OR "Primary Adrenal Insufficiency"[Title/Abstract] OR "adrenal insufficiency primary"[Title/Abstract] OR "Primary Adrenocortical Insufficiency"[Title/Abstract] OR "adrenocortical insufficiency primary"[Title/Abstract] OR "insufficiency primary adrenocortical"[Title/Abstract] OR "Primary Hypoadrenalism"[Title/Abstract] OR "Cystic Fibrosis"[MeSH Terms] OR "fibrosis cystic"[Title/Abstract] OR "Mucoviscidosis"[Title/Abstract] OR "Pancreatic Cystic Fibrosis"[Title/Abstract] OR "cystic fibrosis pancreatic"[Title/Abstract] OR "Cystic Fibrosis of Pancreas"[Title/Abstract] OR "Fibrocystic Disease of Pancreas"[Title/Abstract] OR "Pancreas Fibrocystic Disease"[Title/Abstract] OR "Pulmonary Cystic Fibrosis"[Title/Abstract] OR "cystic fibrosis pulmonary"[Title/Abstract] OR "Thyroid Diseases"[MeSH Terms] OR "diseases thyroid"[Title/Abstract] OR "disease thyroid"[Title/Abstract] OR "Thyroid Disease"[Title/Abstract] OR "Hyperthyroidism"[MeSH Terms] OR "Hyperthyroid"[Title/Abstract] OR "Hyperthyroids"[Title/Abstract] OR "Primary Hyperthyroidism"[Title/Abstract] OR "hyperthyroidism primary"[Title/Abstract] OR "Hypothyroidism"[MeSH Terms] OR "Hypothyroidisms"[Title/Abstract] OR "thyroid stimulating hormone deficiency"[Title/Abstract] OR "deficiency thyroid stimulating hormone"[Title/Abstract] OR "hormone deficiency thyroid stimulating"[Title/Abstract] OR "Thyroid-Stimulating Hormone Deficiencies"[Title/Abstract] OR "thyroid stimulating hormone deficiency"[Title/Abstract] OR "TSH Deficiency"[Title/Abstract] OR "deficiency tsh"[Title/Abstract] OR "TSH Deficiencies"[Title/Abstract] OR "Secondary Hypothyroidism"[Title/Abstract] OR "hypothyroidism secondary"[Title/Abstract] OR "Central Hypothyroidism"[Title/Abstract] OR "hypothyroidism central"[Title/Abstract] OR "Primary Hypothyroidism"[Title/Abstract] OR "hypothyroidism primary"[Title/Abstract] OR "Primary Hypothyroidisms"[Title/Abstract] OR "pulmonary disease, chronic obstructive"[MeSH Terms] OR "Chronic Obstructive Pulmonary Diseases"[Title/Abstract] OR "COPD"[Title/Abstract] OR "Chronic Obstructive Lung Disease"[Title/Abstract] OR "Chronic Obstructive Pulmonary Disease"[Title/Abstract] OR "COAD"[Title/Abstract] OR "Chronic Obstructive Airway Disease"[Title/Abstract] OR "airflow obstruction chronic"[Title/Abstract] OR "airflow obstructions chronic"[Title/Abstract] OR "Chronic Airflow Obstructions"[Title/Abstract] OR "Chronic Airflow Obstruction"[Title/Abstract] OR "Asthma"[MeSH Terms] OR "asthma*"[Title/Abstract] OR "asthma bronchial"[Title/Abstract] OR "Bronchial Asthma"[Title/Abstract] OR "Bronchiectasis"[MeSH Terms] OR "Bronchiectases"[Title/Abstract] OR "Cylindrical Bronchiectasis"[Title/Abstract] OR "bronchiectasis cylindrical"[Title/Abstract] OR "Varicose Bronchiectasis"[Title/Abstract] OR "Varicose Bronchiectases"[Title/Abstract] OR "Saccular Bronchiectasis"[Title/Abstract] OR "bronchiectasis saccular"[Title/Abstract] OR "Cystic Bronchiectasis"[Title/Abstract] OR "bronchiectasis cystic"[Title/Abstract] OR "Cystic Bronchiectases"[Title/Abstract]) AND "ischemic attack, transient"[MeSH Terms]) OR "Brain TIA"[Title/Abstract] OR "tia brain"[Title/Abstract] OR "TIA"[Title/Abstract] OR "Transient Ischemic Attack"[Title/Abstract] OR "attacks transient ischemic"[Title/Abstract] OR "attack transient ischemic"[Title/Abstract] OR "ischemic attacks transient"[Title/Abstract] OR "Transient Ischemic Attacks"[Title/Abstract] OR "cerebral ischemia transient"[Title/Abstract] OR "Transient Cerebral Ischemia"[Title/Abstract] OR "Transient Cerebral Ischemias"[Title/Abstract] OR "transient ischemic attack brainstem"[Title/Abstract] OR "Transient Brainstem Ischemia"[Title/Abstract] OR "Brainstem Transient Ischemic Attack"[Title/Abstract] OR "Crescendo Transient Ischemic Attacks"[Title/Abstract] OR "Posterior Circulation Transient Ischemic Attack"[Title/Abstract] OR "Anterior Circulation Transient Ischemic Attack"[Title/Abstract] OR "Peripheral Nervous System Diseases"[MeSH Terms] OR "Peripheral Nervous System Disorders"[Title/Abstract] OR "Peripheral Neuropathies"[Title/Abstract] OR "neuropathy peripheral"[Title/Abstract] OR "Peripheral Neuropathy"[Title/Abstract] OR "Peripheral Nervous System Disease"[Title/Abstract] OR "PNS Diseases"[Title/Abstract] OR "PNS Disease"[Title/Abstract] OR "Peripheral Nerve Diseases"[Title/Abstract] OR "nerve disease peripheral"[Title/Abstract] OR "Peripheral Nerve Disease"[Title/Abstract] OR "Chronic Pain"[MeSH Terms] OR "pain chronic"[Title/Abstract] OR "Widespread Chronic Pain"[Title/Abstract] OR "chronic pain widespread"[Title/Abstract] OR "Chronic Primary Pain"[Title/Abstract] OR "pain chronic primary"[Title/Abstract] OR "primary pain chronic"[Title/Abstract] OR "Chronic Secondary Pain"[Title/Abstract] OR "pain chronic secondary"[Title/Abstract] OR "secondary pain chronic"[Title/Abstract]) AND "Neoplasms"[MeSH Terms]) OR "Tumors"[Title/Abstract] OR "Neoplasia"[Title/Abstract] OR "Neoplasias"[Title/Abstract] OR "Neoplasm"[Title/Abstract] OR "Tumor"[Title/Abstract] OR "Cancer"[Title/Abstract] OR "Cancers"[Title/Abstract] OR "Malignant Neoplasm"[Title/Abstract] OR "Malignancy"[Title/Abstract] OR "Malignancies"[Title/Abstract] OR "Malignant Neoplasms"[Title/Abstract] OR "neoplasm malignant"[Title/Abstract] OR "neoplasms malignant"[Title/Abstract] OR "Benign Neoplasms"[Title/Abstract] OR "neoplasms benign"[Title/Abstract] OR "neoplasm benign"[Title/Abstract] OR "Benign Neoplasm"[Title/Abstract]) AND "Bone Neoplasms"[MeSH Terms]) OR "Bone Neoplasm"[Title/Abstract] OR "neoplasm bone"[Title/Abstract] OR "neoplasms bone"[Title/Abstract] OR "Bone Cancer"[Title/Abstract] OR "Cancer of Bone"[Title/Abstract] OR "Cancer of the Bone"[Title/Abstract] OR "Melanoma"[MeSH Terms] OR "Melanomas"[Title/Abstract] OR "Malignant Melanoma"[Title/Abstract] OR "Malignant Melanomas"[Title/Abstract] OR "melanoma malignant"[Title/Abstract] OR "melanomas malignant"[Title/Abstract]) AND "Dementia"[MeSH Terms]) OR "Dementias"[Title/Abstract] OR "Amentia"[Title/Abstract] OR "Amentias"[Title/Abstract] OR "Familial Dementia"[Title/Abstract] OR "dementia familial"[Title/Abstract] OR "dementias familial"[Title/Abstract] OR "Familial Dementias"[Title/Abstract] OR "Schizophrenia"[MeSH Terms] OR "schizophrenia*"[Title/Abstract] OR "Dementia Praecox"[Title/Abstract] OR "Schizophrenic Disorders"[Title/Abstract] OR "disorder schizophrenic"[Title/Abstract] OR "disorders schizophrenic"[Title/Abstract] OR "Schizophrenic Disorder"[Title/Abstract] OR "Depression"[MeSH Terms] OR "Depressive Symptoms"[Title/Abstract] OR "Depressive Symptom"[Title/Abstract] OR "symptom depressive"[Title/Abstract] OR "Emotional Depression"[Title/Abstract] OR "depression emotional"[Title/Abstract] OR "Depressive Disorder"[MeSH Terms] OR "Depressive Disorders"[Title/Abstract] OR "disorder depressive"[Title/Abstract] OR "disorders depressive"[Title/Abstract] OR "neurosis depressive"[Title/Abstract] OR "Depressive Neuroses"[Title/Abstract] OR "Depressive Neurosis"[Title/Abstract] OR "neuroses depressive"[Title/Abstract] OR "depression endogenous"[Title/Abstract] OR "depressions endogenous"[Title/Abstract] OR "Endogenous Depression"[Title/Abstract] OR "Endogenous Depressions"[Title/Abstract] OR "melancholia*"[Title/Abstract] OR "Unipolar Depression"[Title/Abstract] OR "depression unipolar"[Title/Abstract] OR "depressions unipolar"[Title/Abstract] OR "Unipolar Depressions"[Title/Abstract] OR "Depressive Syndrome"[Title/Abstract] OR "Depressive Syndromes"[Title/Abstract] OR "syndrome depressive"[Title/Abstract] OR "syndromes depressive"[Title/Abstract] OR "depression neurotic"[Title/Abstract] OR "Neurotic Depression"[Title/Abstract] OR "Neurotic Depressions"[Title/Abstract] OR "Anxiety"[MeSH Terms] OR "Angst"[Title/Abstract] OR "Nervousness"[Title/Abstract] OR "Hypervigilance"[Title/Abstract] OR "Social Anxiety"[Title/Abstract] OR "anxiety social"[Title/Abstract] OR "Social Anxieties"[Title/Abstract] OR "Anxiousness"[Title/Abstract] OR "Bipolar Disorder"[MeSH Terms] OR "Bipolar Disorders"[Title/Abstract] OR "disorder bipolar"[Title/Abstract] OR "Bipolar Mood Disorder"[Title/Abstract] OR "Bipolar Mood Disorders"[Title/Abstract] OR "disorder bipolar mood"[Title/Abstract] OR "mood disorder bipolar"[Title/Abstract] OR "affective psychosis bipolar"[Title/Abstract] OR "Bipolar Affective Psychosis"[Title/Abstract] OR "psychoses bipolar affective"[Title/Abstract] OR "psychosis bipolar affective"[Title/Abstract] OR "psychoses manic depressive"[Title/Abstract] OR "psychoses manic depressive"[Title/Abstract] OR "manic depressive psychosis"[Title/Abstract] OR "manic depressive psychosis"[Title/Abstract] OR "psychosis manic depressive"[Title/Abstract] OR "psychosis manic depressive"[Title/Abstract] OR "depression bipolar"[Title/Abstract] OR "Bipolar Depression"[Title/Abstract] OR "Manic Disorder"[Title/Abstract] OR "disorder manic"[Title/Abstract] OR "Manic Disorders"[Title/Abstract] OR "Manic Depression"[Title/Abstract] OR "depression manic"[Title/Abstract] OR "depressions manic"[Title/Abstract] OR "Bipolar Disorder Type 1"[Title/Abstract] OR "Type 1 Bipolar Disorder"[Title/Abstract] OR "Bipolar Disorder Type 2"[Title/Abstract] OR "Type 2 Bipolar Disorder"[Title/Abstract] OR "Drug Misuse"[MeSH Terms] OR "Drug Misuses"[Title/Abstract] OR "misuse drug"[Title/Abstract] OR "non prescription medicine misuse"[Title/Abstract] OR "non prescription medicine misuse"[Title/Abstract] OR "Nonprescription Drug Misuse"[Title/Abstract] OR "over the counter drug misuse"[Title/Abstract] OR "misuse over the counter drug"[Title/Abstract] OR "over the counter drug misuse"[Title/Abstract] OR "Feeding and Eating Disorders"[MeSH Terms] OR "Eating and Feeding Disorders"[Title/Abstract] OR "Appetite Disorders"[Title/Abstract] OR "Appetite Disorder"[Title/Abstract] OR "Eating Disorders"[Title/Abstract] OR "disorder eating"[Title/Abstract] OR "disorders eating"[Title/Abstract] OR "Eating Disorder"[Title/Abstract] OR "Feeding Disorders"[Title/Abstract] OR "disorder feeding"[Title/Abstract] OR "disorders feeding"[Title/Abstract] OR "Feeding Disorder"[Title/Abstract] OR "Autistic Disorder"[MeSH Terms] OR "disorder autistic"[Title/Abstract] OR "disorders autistic"[Title/Abstract] OR "Autism"[Title/Abstract] OR "autism early infantile"[Title/Abstract] OR "Early Infantile Autism"[Title/Abstract] OR "infantile autism early"[Title/Abstract] OR "autism infantile"[Title/Abstract] OR "Infantile Autism"[Title/Abstract] OR "Kanner's Syndrome"[Title/Abstract] OR "Kanners Syndrome"[Title/Abstract] OR "Kanner Syndrome"[Title/Abstract] OR "stress disorders, post traumatic"[MeSH Terms] OR "post traumatic stress disorder"[Title/Abstract] OR "stress disorder post traumatic"[Title/Abstract] OR "post traumatic stress disorder"[Title/Abstract] OR "neuroses post traumatic"[Title/Abstract] OR "neuroses post traumatic"[Title/Abstract] OR "Post-Traumatic Neuroses"[Title/Abstract] OR "PTSD"[Title/Abstract] OR "stress disorder post traumatic"[Title/Abstract] OR "post traumatic stress disorders"[Title/Abstract] OR "post traumatic stress disorders"[Title/Abstract] OR "Posttraumatic Stress Disorders"[Title/Abstract] OR "Posttraumatic Stress Disorder"[Title/Abstract] OR "stress disorder posttraumatic"[Title/Abstract] OR "stress disorders posttraumatic"[Title/Abstract] OR "Posttraumatic Neuroses"[Title/Abstract] OR "acute post traumatic stress disorder"[Title/Abstract] OR "acute post traumatic stress disorder"[Title/Abstract] OR "chronic post traumatic stress disorder"[Title/Abstract] OR "chronic post traumatic stress disorder"[Title/Abstract] OR "delayed onset post traumatic stress disorder"[Title/Abstract] OR "delayed onset post traumatic stress disorder"[Title/Abstract] OR "Moral Injury"[Title/Abstract] OR "injury moral"[Title/Abstract] OR "Moral Injuries"[Title/Abstract]) AND "Musculoskeletal Diseases"[MeSH Terms]) OR "Musculoskeletal Disease"[Title/Abstract] OR "orthopedic disorder*"[Title/Abstract] OR "orthopaedic disorder*"[Title/Abstract] OR "Connective Tissue Diseases"[MeSH Terms] OR "Connective Tissue Disease"[Title/Abstract] OR "disease connective tissue"[Title/Abstract] OR "diseases connective tissue"[Title/Abstract] OR "Arthritis"[MeSH Terms] OR "Arthritides"[Title/Abstract] OR "Polyarthritis"[Title/Abstract] OR "Polyarthritides"[Title/Abstract] OR "Oligoarthritis"[Title/Abstract] OR "Oligoarthritides"[Title/Abstract] OR "Osteoarthritis"[MeSH Terms] OR "Osteoarthritides"[Title/Abstract] OR "arthritis degenerative"[Title/Abstract] OR "Degenerative Arthritides"[Title/Abstract] OR "Degenerative Arthritis"[Title/Abstract] OR "Osteoarthrosis"[Title/Abstract] OR "Osteoarthroses"[Title/Abstract] OR "Osteoarthrosis Deformans"[Title/Abstract] OR "Arthrosis"[Title/Abstract] OR "Arthroses"[Title/Abstract] OR "Osteoporosis"[MeSH Terms] OR "Osteoporoses"[Title/Abstract] OR "Osteoporosis"[Title/Abstract] OR "osteoporosis age related"[Title/Abstract] OR "osteoporosis age related"[Title/Abstract] OR "age related osteoporosis"[Title/Abstract] OR "Age-Related Osteoporoses"[Title/Abstract] OR "age related osteoporosis"[Title/Abstract] OR "bone loss age related"[Title/Abstract] OR "Age-Related Bone Loss"[Title/Abstract] OR "Age-Related Bone Losses"[Title/Abstract] OR "bone loss age related"[Title/Abstract] OR "osteoporosis senile"[Title/Abstract] OR "Senile Osteoporosis"[Title/Abstract] OR "osteoporosis post traumatic"[Title/Abstract] OR "osteoporosis post traumatic"[Title/Abstract] OR "Post-Traumatic Osteoporosis"[Title/Abstract] OR "Gout"[MeSH Terms] OR "gout*"[Title/Abstract] OR "chronic digestive disease"[Title/Abstract] OR "chronic gastrointestinal disease"[Title/Abstract] OR "chronic GI disorders"[Title/Abstract] OR "chronic intestinal diseases"[Title/Abstract] OR "gastroesophageal reflux disease"[Title/Abstract] OR "GERD"[Title/Abstract] OR "chronic irritable bowel syndrome"[Title/Abstract] OR "Crohn's disease"[Title/Abstract] OR "chronic ulcerative colitis"[Title/Abstract] OR "Celiac Disease"[Title/Abstract] OR "chronic peptic ulcer"[Title/Abstract] OR "chronic diverticulitis"[Title/Abstract] OR "chronic gallbladder disease"[Title/Abstract] OR "chronic liver disease"[Title/Abstract] OR "chronic pancreatitis"[Title/Abstract] OR "chronic abdominal pain"[Title/Abstract] OR "chronic diarrhea"[Title/Abstract] OR "chronic constipation"[Title/Abstract] OR "chronic dyspepsia"[Title/Abstract] OR "chronic nausea and vomiting"[Title/Abstract] OR "chronic dysbiosis"[Title/Abstract] OR "chronic motility disorders"[Title/Abstract] OR "chronic malabsorption syndromes"[Title/Abstract] OR "Gastroesophageal Reflux"[MeSH Terms] OR "Irritable Bowel Syndrome"[MeSH Terms] OR "Crohn Disease"[MeSH Terms] OR "Celiac Disease"[MeSH Terms] OR "End Stage Liver Disease"[MeSH Terms] OR "pancreatitis, chronic"[MeSH Terms] OR "Ciliary Motility Disorders"[MeSH Terms] OR "chronic kidney disease"[Title/Abstract] OR "end-stage kidney disease"[Title/Abstract] OR "end-stage renal disease"[Title/Abstract] OR "chronic renal failure"[Title/Abstract] OR "Renal Insufficiency"[Title/Abstract] OR "dialysis-dependent kidney disease"[Title/Abstract] OR "Endometriosis"[Title/Abstract] OR "deep infiltrating endometriosis"[Title/Abstract] OR "chronic pelvic pain"[Title/Abstract] OR "endometriosis-associated infertility"[Title/Abstract] OR "chronic urinary tract infection"[Title/Abstract] OR "recurrent urinary tract infection"[Title/Abstract] OR "interstitial cystitis"[Title/Abstract] OR "bladder pain syndrome"[Title/Abstract] OR "kidney failure, chronic"[MeSH Terms] OR "Renal Insufficiency"[MeSH Terms] OR "Endometriosis"[MeSH Terms] OR "cystitis, interstitial"[MeSH Terms] OR "Thalassemia"[Title/Abstract] OR "sickle cell disease"[Title/Abstract] OR "Polycythemia Vera"[Title/Abstract] OR "essential thrombocythemia"[Title/Abstract] OR "Myelodysplastic Syndromes"[Title/Abstract] OR "chronic myeloid leukemia"[Title/Abstract] OR "chronic lymphocytic leukemia"[Title/Abstract] OR "Lymphoma"[Title/Abstract] OR "Hodgkin lymphoma"[Title/Abstract] OR "non-Hodgkin lymphoma"[Title/Abstract] OR "Multiple Myeloma"[Title/Abstract] OR "Hemochromatosis"[Title/Abstract] OR "paroxysmal nocturnal hemoglobinuria"[Title/Abstract] OR "beta thalassemia"[Title/Abstract] OR "alpha thalassemia"[Title/Abstract] OR "Hemoglobinopathies"[Title/Abstract] OR "plasma cell dyscrasias"[Title/Abstract] OR "Thalassemia"[MeSH Terms] OR "anemia, sickle cell"[MeSH Terms] OR "Polycythemia Vera"[MeSH Terms] OR "thrombocythemia, essential"[MeSH Terms] OR "Myelodysplastic Syndromes"[MeSH Terms] OR "Leukemia"[MeSH Terms] OR "Lymphoma"[MeSH Terms] OR "Hodgkin Disease"[MeSH Terms] OR "Multiple Myeloma"[MeSH Terms] OR "Hemochromatosis"[MeSH Terms] OR "hemoglobinuria, paroxysmal"[MeSH Terms] OR "beta thalassemia"[MeSH Terms] OR "alpha thalassemia"[MeSH Terms] OR "Hemoglobinopathies"[MeSH Terms] OR "Paraproteinemias"[MeSH Terms] OR "irreversible vision loss"[Title/Abstract] OR "Blindness"[Title/Abstract] OR "age-related macular degeneration"[Title/Abstract] OR "ARMD"[Title/Abstract] OR "Retinitis Pigmentosa"[Title/Abstract] OR "Glaucoma"[Title/Abstract] OR "Diabetic Retinopathy"[Title/Abstract] OR "Cataract"[Title/Abstract] OR "optic neuropathy"[Title/Abstract] OR "Uveitis"[Title/Abstract] OR "corneal scarring"[Title/Abstract] OR "albinism"[Title/Abstract] OR "leber congenital amaurosis"[Title/Abstract] OR "Optic Atrophy"[Title/Abstract] OR "Retinal Detachment"[Title/Abstract] OR "macular hole"[Title/Abstract] OR "dry eye"[Title/Abstract] OR "Aniridia"[Title/Abstract] OR "Choroideremia"[Title/Abstract] OR "Stargardt Disease"[Title/Abstract] OR "fuchs dystrophy"[Title/Abstract] OR "cone-rod dystrophy"[Title/Abstract] OR "nystagmus"[Title/Abstract] OR "Amblyopia"[Title/Abstract] OR "visual field loss"[Title/Abstract] OR "Blindness"[MeSH Terms] OR "Retinitis Pigmentosa"[MeSH Terms] OR "Glaucoma"[MeSH Terms] OR "Diabetic Retinopathy"[MeSH Terms] OR "Cataract"[MeSH Terms] OR "optic neuropathy, ischemic"[MeSH Terms] OR "Uveitis"[MeSH Terms] OR "Corneal Injuries"[MeSH Terms] OR "albinism, ocular"[MeSH Terms] OR "leber congenital amaurosis"[MeSH Terms] OR "Optic Atrophy"[MeSH Terms] OR "Retinal Detachment"[MeSH Terms] OR "Retinal Diseases"[MeSH Terms] OR "Dry Eye Syndromes"[MeSH Terms] OR "Aniridia"[MeSH Terms] OR "Choroideremia"[MeSH Terms] OR "Stargardt Disease"[MeSH Terms] OR "Cone-Rod Dystrophies"[MeSH Terms] OR "nystagmus, congenital"[MeSH Terms] OR "Amblyopia"[MeSH Terms]) AND "Meniere's disease"[Title/Abstract]) OR "sensorineural hearing loss"[Title/Abstract] OR "congenital deafness"[Title/Abstract] OR "age-related hearing loss"[Title/Abstract] OR "Presbycusis"[Title/Abstract] OR "noise-induced hearing loss"[Title/Abstract] OR "Ototoxicity"[Title/Abstract] OR "Meniere's disease"[Title/Abstract] OR "genetic hearing loss"[Title/Abstract] OR "autoimmune inner ear disease"[Title/Abstract] OR "auditory neuropathy spectrum disorder"[Title/Abstract] OR "Usher syndrome"[Title/Abstract] OR "waardenburg syndrome"[Title/Abstract] OR "Pendred syndrome"[Title/Abstract] OR "Alport syndrome"[Title/Abstract] OR "labyrinthitis"[Title/Abstract] OR "viral cochleitis"[Title/Abstract] OR "acoustic neuroma"[Title/Abstract] OR "bilateral vestibular schwannoma"[Title/Abstract] OR "Cholesteatoma"[Title/Abstract] OR "perilymph fistula"[Title/Abstract] OR "trauma-induced hearing loss"[Title/Abstract] OR "permanent hearing loss"[Title/Abstract] OR "irreversible hearing loss"[Title/Abstract] OR "sensorineural hearing loss"[Title/Abstract] OR "chronic hearing impairment"[Title/Abstract] OR "sensorineural deafness"[Title/Abstract] OR "permanent deafness"[Title/Abstract] OR "hearing loss rehabilitation"[Title/Abstract] OR "hearing loss management"[Title/Abstract] OR "congenital hearing impairment"[Title/Abstract] OR "Deafness"[MeSH Terms] OR "hearing loss, sensorineural"[MeSH Terms] OR "deafness congenital with inner ear agenesis microtia and microdontia"[Supplementary Concept] OR "Presbycusis"[MeSH Terms] OR "hearing loss, noise induced"[MeSH Terms] OR "Ototoxicity"[MeSH Terms] OR "Meniere Disease"[MeSH Terms] OR "Usher Syndromes"[MeSH Terms] OR "waardenburg syndrome"[MeSH Terms] OR "Pendred syndrome"[Supplementary Concept] OR "neuroma, acoustic"[MeSH Terms] OR "Cholesteatoma"[MeSH Terms] OR "HIV"[Title/Abstract] OR "Human Immunodeficiency Virus"[Title/Abstract] OR "AIDS"[Title/Abstract] OR "Acquired Immunodeficiency Syndrome"[Title/Abstract] OR "HIV Infection"[Title/Abstract] OR "HIV Disease"[Title/Abstract] OR "AIDS Virus"[Title/Abstract] OR "HIV Positive"[Title/Abstract] OR "HIV/AIDS"[Title/Abstract] OR "HIV/AIDS Infections"[Title/Abstract] OR "HIV"[MeSH Terms] OR "Acquired Immunodeficiency Syndrome"[MeSH Terms] OR "Tuberculosis"[MeSH Terms] OR "Tuberculosis"[Title/Abstract] OR "TB"[Title/Abstract] OR "chronic Lyme disease"[Title/Abstract] OR "post-treatment Lyme disease syndrome"[Title/Abstract] OR "post-Lyme disease"[Title/Abstract] OR "chronic symptoms of Lyme disease"[Title/Abstract] OR "persistent Lyme disease"[Title/Abstract] OR "Post-Lyme Disease Syndrome"[MeSH Terms] OR "post-acute COVID-19"[Title/Abstract] OR "long COVID"[Title/Abstract] OR "post-COVID syndrome"[Title/Abstract] OR "chronic COVID-19 symptoms"[Title/Abstract] OR "COVID-19 long-term effects"[Title/Abstract] OR "post-viral fatigue syndrome"[Title/Abstract] OR "persistent COVID symptoms"[Title/Abstract] OR "post-acute sequelae of SARS-CoV-2 infection"[Title/Abstract] OR "post-COVID-19 rehabilitation"[Title/Abstract] OR "Post-Acute COVID-19 Syndrome"[MeSH Terms] OR "COVID-19 post-intensive care syndrome"[Supplementary Concept] OR "Congenital Disorders"[Title/Abstract] OR "Congenital Anomalies"[Title/Abstract] OR "Congenital Malformations"[Title/Abstract] OR "Birth Defects"[Title/Abstract] OR "Prenatal Abnormalities"[Title/Abstract] OR "Genetic Birth Defects"[Title/Abstract] OR "Inborn Errors of Metabolism"[Title/Abstract] OR "Congenital Syndromes"[Title/Abstract] OR "Congenital Heart Defects"[Title/Abstract] OR "Neonatal Disorders"[Title/Abstract] OR "Congenital Brain Abnormalities"[Title/Abstract] OR "Congenital Skeletal Abnormalities"[Title/Abstract] OR "Neonatal Genetic Disorders"[Title/Abstract] OR "Prenatal Diagnoses"[Title/Abstract] OR "Embryonic Development Disorders"[Title/Abstract] OR "Chromosomal Disorders"[Title/Abstract] OR "Chromosomal Abnormalities"[Title/Abstract] OR "Chromosome Disorders"[Title/Abstract] OR "Chromosome Aberrations"[Title/Abstract] OR "Down Syndrome"[Title/Abstract] OR "Klinefelter Syndrome"[Title/Abstract] OR "Turner Syndrome"[Title/Abstract] OR "Patau Syndrome"[Title/Abstract] OR "Edward Syndrome"[Title/Abstract] OR "Trisomy Disorders"[Title/Abstract] OR "Structural Chromosome Abnormalities"[Title/Abstract] OR "Balanced Chromosome Translocation"[Title/Abstract] OR "Unbalanced Chromosome Aberrations"[Title/Abstract] OR "Cytogenetic Abnormalities"[Title/Abstract] OR "Autosomal Dominant Inheritance"[Title/Abstract] OR "Autosomal Recessive Disorders"[Title/Abstract] OR "X-linked Diseases"[Title/Abstract] OR "Mitochondrial Genetic Disorders"[Title/Abstract] OR "Genetic Abnormalities"[Title/Abstract] OR "Inherited Disorders"[Title/Abstract] OR "Genomic Disorders"[Title/Abstract] OR "Genetic Counseling"[Title/Abstract] OR "congenital, hereditary, and neonatal diseases and abnormalities"[MeSH Terms])

**#2 Concept (C): engagement and involvement**

"community based participatory research"[Title/Abstract] OR "Participatory Research Community-Based"[Title/Abstract] OR "consumer driven community based research"[Title/Abstract] OR "consumer driven community based research"[Title/Abstract] OR "consumer driven community based research"[Title/Abstract] OR "Participation Community"[Title/Abstract] OR "community involv*"[Title/Abstract] OR "Involvement Community"[Title/Abstract] OR "community action*"[Title/Abstract] OR "Action Community"[Title/Abstract] OR "Consumer Participation"[Title/Abstract] OR "Participation Consumer"[Title/Abstract] OR "consumer involv*"[Title/Abstract] OR "Involvement Consumer"[Title/Abstract] OR "Public Participation"[Title/Abstract] OR "Participation Public"[Title/Abstract] OR "stakeholder role*"[Title/Abstract] OR "stakeholder engagement*"[Title/Abstract] OR "Engagement Stakeholder"[Title/Abstract] OR "participa*"[Title/Abstract] OR "Participation"[Title/Abstract] OR "Participation Patient"[Title/Abstract] OR "Patient Involvement"[Title/Abstract] OR "Involvement Patient"[Title/Abstract] OR "Patient Participation Rates"[Title/Abstract] OR "Participation Rates Patient"[Title/Abstract] OR "Patient Participation Rate"[Title/Abstract] OR "Patient Engagement"[Title/Abstract] OR "Engagement Patient"[Title/Abstract] OR "Patient Activation"[Title/Abstract] OR "Activation Patient"[Title/Abstract] OR "Patient Empowerment"[Title/Abstract] OR "Empowerment Patient"[Title/Abstract] OR "Participant involvement"[Title/Abstract] OR "Participation Community"[Title/Abstract] OR "community involvement*"[Title/Abstract] OR "involvement* community"[Title/Abstract] OR "community action*"[Title/Abstract] OR "Action Community"[Title/Abstract] OR "Actions Community"[Title/Abstract] OR "Consumer Participation"[Title/Abstract] OR "Participation Consumer"[Title/Abstract] OR "consumer involv*"[Title/Abstract] OR "involv* consumer"[Title/Abstract] OR "Public Participation"[Title/Abstract] OR "Participation Public"[Title/Abstract] OR "participatory action research"[Title/Abstract] OR "Community engagement"[Title/Abstract] OR "community involve*"[Title/Abstract] OR "Community Participation"[Title/Abstract] OR "Patient Participation"[Title/Abstract] OR "Stakeholder Participation"[Title/Abstract] OR "community based participatory research"[MeSH Terms] OR "Community Participation"[MeSH Terms] OR "Patient Participation"[MeSH Terms] OR "Stakeholder Participation"[MeSH Terms]

**#3 Context (C): Theories OR Models OR Frameworks 10496**

"theory of change"[Title/Abstract] OR "participatory model"[Title/Abstract] OR "shared decision-making model"[Title/Abstract] OR "engagement framework"[Title/Abstract] OR "involvement framework"[Title/Abstract] OR "patient-centered care model"[Title/Abstract] OR "self-management framework"[Title/Abstract] OR "chronic care model"[Title/Abstract] OR "community engagement model"[Title/Abstract] OR "co-production model"[Title/Abstract] OR "co-design framework"[Title/Abstract] OR "social-ecological model"[Title/Abstract] OR "health belief model"[Title/Abstract] OR "behavioral change theory"[Title/Abstract] OR "transtheoretical model"[Title/Abstract] OR "collaborative care model"[Title/Abstract] OR "empowerment theory"[Title/Abstract] OR "person-centered care framework"[Title/Abstract]

**#1 AND #2 AND #3 534**

("multiple comorbidity"[Title/Abstract] OR "multimorb*"[Title/Abstract] OR "multimorbid*"[Title/Abstract] OR "multimorbidit*"[Title/Abstract] OR "multi morbidit*"[Title/Abstract] OR "multi morbidit*"[Title/Abstract] OR "multi disease*"[Title/Abstract] OR "multi morbid*"[Title/Abstract] OR "multidisease"[Title/Abstract] OR "multiple diseases"[Title/Abstract] OR "multi-disease"[Title/Abstract] OR "multicondition*"[Title/Abstract] OR "multi condition*"[Title/Abstract] OR "multiple condition"[Title/Abstract] OR "multisyndrome*"[Title/Abstract] OR "multi-syndrome"[Title/Abstract] OR "multiple syndrome"[Title/Abstract] OR "multidisorder*"[Title/Abstract] OR "multi-disorder"[Title/Abstract] OR "multiple disorder"[Title/Abstract] OR "multiproblem*"[Title/Abstract] OR "multiple chronic condition*"[Title/Abstract] OR "morbidity burden*"[Title/Abstract] OR "multipatholog*"[Title/Abstract] OR "polypatholog*"[Title/Abstract] OR "comorb*"[Title/Abstract] OR "comorbid*"[Title/Abstract] OR "co morbid*"[Title/Abstract] OR "co morbid*"[Title/Abstract] OR "comorbidit*"[Title/Abstract] OR "co morbidit*"[Title/Abstract] OR "noncommunicable"[Title/Abstract] OR "non communicable"[Title/Abstract] OR "polymorb*"[Title/Abstract] OR "polymorbidit*"[Title/Abstract] OR "poly morbidit*"[Title/Abstract] OR "pluripatholog*"[Title/Abstract] OR "pluri patholog*"[Title/Abstract] OR "Multimorbidity"[MeSH Terms] OR "Multiple Chronic Conditions"[MeSH Terms] OR ((((((((((((("Cardiovascular Diseases"[MeSH Terms] OR "Cardiovascular Disease"[Title/Abstract] OR "disease cardiovascular"[Title/Abstract] OR "Cardiac Events"[Title/Abstract] OR "Cardiac Event"[Title/Abstract] OR "event cardiac"[Title/Abstract] OR "Adverse Cardiac Event"[Title/Abstract] OR "Adverse Cardiac Events"[Title/Abstract] OR "cardiac events adverse"[Title/Abstract] OR "Major Adverse Cardiac Events"[Title/Abstract] OR "Stroke"[MeSH Terms] OR "Stroke"[Title/Abstract] OR "Strokes"[Title/Abstract] OR "Cerebrovascular Accident"[Title/Abstract] OR "Cerebrovascular Accidents"[Title/Abstract] OR "Cerebral Stroke"[Title/Abstract] OR "Cerebral Strokes"[Title/Abstract] OR "stroke cerebral"[Title/Abstract] OR "strokes cerebral"[Title/Abstract] OR "Cerebrovascular Apoplexy"[Title/Abstract] OR "apoplexy cerebrovascular"[Title/Abstract] OR "vascular accident brain"[Title/Abstract] OR "Brain Vascular Accident"[Title/Abstract] OR "Brain Vascular Accidents"[Title/Abstract] OR "Cerebrovascular Stroke"[Title/Abstract] OR "Cerebrovascular Strokes"[Title/Abstract] OR "stroke cerebrovascular"[Title/Abstract] OR "strokes cerebrovascular"[Title/Abstract] OR "Apoplexy"[Title/Abstract] OR "CVA"[Title/Abstract] OR "CVA"[Title/Abstract] OR "stroke acute"[Title/Abstract] OR "Acute Stroke"[Title/Abstract] OR "Acute Strokes"[Title/Abstract] OR "strokes acute"[Title/Abstract] OR "cerebrovascular accident acute"[Title/Abstract] OR "Acute Cerebrovascular Accident"[Title/Abstract] OR "Acute Cerebrovascular Accidents"[Title/Abstract] OR "cerebrovascular accidents acute"[Title/Abstract] OR "Coronary Artery Disease"[MeSH Terms] OR "Coronary Disease"[MeSH Terms] OR "artery disease coronary"[Title/Abstract] OR "artery diseases coronary"[Title/Abstract] OR "Coronary Artery Diseases"[Title/Abstract] OR "Coronary Arteriosclerosis"[Title/Abstract] OR "arteriosclerosis coronary"[Title/Abstract] OR "atherosclerosis coronary"[Title/Abstract] OR "Coronary Atherosclerosis"[Title/Abstract] OR "Left Main Coronary Artery Disease"[Title/Abstract] OR "Left Main Coronary Disease"[Title/Abstract] OR "Left Main Disease"[Title/Abstract] OR "Left Main Diseases"[Title/Abstract] OR "Heart Failure"[MeSH Terms] OR "Cardiac Failure"[Title/Abstract] OR "Heart Decompensation"[Title/Abstract] OR "decompensation heart"[Title/Abstract] OR "Congestive Heart Failure"[Title/Abstract] OR "heart failure congestive"[Title/Abstract] OR "heart failure right sided"[Title/Abstract] OR "heart failure right sided"[Title/Abstract] OR "right sided heart failure"[Title/Abstract] OR "right sided heart failure"[Title/Abstract] OR "heart failure left sided"[Title/Abstract] OR "heart failure left sided"[Title/Abstract] OR "left sided heart failure"[Title/Abstract] OR "left sided heart failure"[Title/Abstract] OR "Myocardial Failure"[Title/Abstract] OR "Peripheral Arterial Disease"[MeSH Terms] OR "arterial disease peripheral"[Title/Abstract] OR "disease peripheral arterial"[Title/Abstract] OR "diseases peripheral arterial"[Title/Abstract] OR "Peripheral Arterial Diseases"[Title/Abstract] OR "Peripheral Artery Disease"[Title/Abstract] OR "artery disease peripheral"[Title/Abstract] OR "artery diseases peripheral"[Title/Abstract] OR "disease peripheral artery"[Title/Abstract] OR "diseases peripheral artery"[Title/Abstract] OR "Peripheral Artery Diseases"[Title/Abstract] OR "Heart Valve Diseases"[MeSH Terms] OR "Heart Valve Disease"[Title/Abstract] OR "valve disease heart"[Title/Abstract] OR "Valvular Heart Diseases"[Title/Abstract] OR "heart disease valvular"[Title/Abstract] OR "Valvular Heart Disease"[Title/Abstract] OR "Heart Valvular Disease"[Title/Abstract] OR "Heart Valvular Diseases"[Title/Abstract] OR "valvular disease heart"[Title/Abstract] OR "arrhythmias, cardiac"[MeSH Terms] OR "arrhythmia cardiac"[Title/Abstract] OR "Arrhythmia"[Title/Abstract] OR "Arrythmia"[Title/Abstract] OR "Cardiac Arrhythmia"[Title/Abstract] OR "Cardiac Arrhythmias"[Title/Abstract] OR "Cardiac Dysrhythmia"[Title/Abstract] OR "dysrhythmia cardiac"[Title/Abstract] OR "Respiratory Sinus Arrhythmia"[MeSH Terms] OR "sinus arrhythmia respiratory"[Title/Abstract] OR "arrhythmia, sinus"[MeSH Terms] OR "arrhythmias sinus"[Title/Abstract] OR "Sinus Arrhythmias"[Title/Abstract] OR "Sinus Arrhythmia"[Title/Abstract] OR "arrhythmias sinoatrial"[Title/Abstract] OR "Sinoatrial Arrhythmias"[Title/Abstract] OR "Sinoatrial Arrhythmia"[Title/Abstract] OR "Thromboembolism"[MeSH Terms] OR "thromboembolism*"[Title/Abstract] OR "Aneurysm"[MeSH Terms] OR "Aneurysms"[Title/Abstract] OR "Fusiform Aneurysm"[Title/Abstract] OR "aneurysm fusiform"[Title/Abstract] OR "aneurysms fusiform"[Title/Abstract] OR "Fusiform Aneurysms"[Title/Abstract] OR "Saccular Aneurysm"[Title/Abstract] OR "Hypertension"[MeSH Terms] OR "blood pressure high"[Title/Abstract] OR "blood pressures high"[Title/Abstract] OR "High Blood Pressure"[Title/Abstract] OR "High Blood Pressures"[Title/Abstract] OR "Diabetes Mellitus"[MeSH Terms] OR "Addison Disease"[MeSH Terms] OR "disease addison"[Title/Abstract] OR "Addison's Disease"[Title/Abstract] OR "Addisons Disease"[Title/Abstract] OR "Primary Adrenal Insufficiency"[Title/Abstract] OR "adrenal insufficiency primary"[Title/Abstract] OR "Primary Adrenocortical Insufficiency"[Title/Abstract] OR "adrenocortical insufficiency primary"[Title/Abstract] OR "insufficiency primary adrenocortical"[Title/Abstract] OR "Primary Hypoadrenalism"[Title/Abstract] OR "Cystic Fibrosis"[MeSH Terms] OR "fibrosis cystic"[Title/Abstract] OR "Mucoviscidosis"[Title/Abstract] OR "Pancreatic Cystic Fibrosis"[Title/Abstract] OR "cystic fibrosis pancreatic"[Title/Abstract] OR "Cystic Fibrosis of Pancreas"[Title/Abstract] OR "Fibrocystic Disease of Pancreas"[Title/Abstract] OR "Pancreas Fibrocystic Disease"[Title/Abstract] OR "Pulmonary Cystic Fibrosis"[Title/Abstract] OR "cystic fibrosis pulmonary"[Title/Abstract] OR "Thyroid Diseases"[MeSH Terms] OR "diseases thyroid"[Title/Abstract] OR "disease thyroid"[Title/Abstract] OR "Thyroid Disease"[Title/Abstract] OR "Hyperthyroidism"[MeSH Terms] OR "Hyperthyroid"[Title/Abstract] OR "Hyperthyroids"[Title/Abstract] OR "Primary Hyperthyroidism"[Title/Abstract] OR "hyperthyroidism primary"[Title/Abstract] OR "Hypothyroidism"[MeSH Terms] OR "Hypothyroidisms"[Title/Abstract] OR "thyroid stimulating hormone deficiency"[Title/Abstract] OR "deficiency thyroid stimulating hormone"[Title/Abstract] OR "hormone deficiency thyroid stimulating"[Title/Abstract] OR "Thyroid-Stimulating Hormone Deficiencies"[Title/Abstract] OR "thyroid stimulating hormone deficiency"[Title/Abstract] OR "TSH Deficiency"[Title/Abstract] OR "deficiency tsh"[Title/Abstract] OR "TSH Deficiencies"[Title/Abstract] OR "Secondary Hypothyroidism"[Title/Abstract] OR "hypothyroidism secondary"[Title/Abstract] OR "Central Hypothyroidism"[Title/Abstract] OR "hypothyroidism central"[Title/Abstract] OR "Primary Hypothyroidism"[Title/Abstract] OR "hypothyroidism primary"[Title/Abstract] OR "Primary Hypothyroidisms"[Title/Abstract] OR "pulmonary disease, chronic obstructive"[MeSH Terms] OR "Chronic Obstructive Pulmonary Diseases"[Title/Abstract] OR "COPD"[Title/Abstract] OR "Chronic Obstructive Lung Disease"[Title/Abstract] OR "Chronic Obstructive Pulmonary Disease"[Title/Abstract] OR "COAD"[Title/Abstract] OR "Chronic Obstructive Airway Disease"[Title/Abstract] OR "airflow obstruction chronic"[Title/Abstract] OR "airflow obstructions chronic"[Title/Abstract] OR "Chronic Airflow Obstructions"[Title/Abstract] OR "Chronic Airflow Obstruction"[Title/Abstract] OR "Asthma"[MeSH Terms] OR "asthma*"[Title/Abstract] OR "asthma bronchial"[Title/Abstract] OR "Bronchial Asthma"[Title/Abstract] OR "Bronchiectasis"[MeSH Terms] OR "Bronchiectases"[Title/Abstract] OR "Cylindrical Bronchiectasis"[Title/Abstract] OR "bronchiectasis cylindrical"[Title/Abstract] OR "Varicose Bronchiectasis"[Title/Abstract] OR "Varicose Bronchiectases"[Title/Abstract] OR "Saccular Bronchiectasis"[Title/Abstract] OR "bronchiectasis saccular"[Title/Abstract] OR "Cystic Bronchiectasis"[Title/Abstract] OR "bronchiectasis cystic"[Title/Abstract] OR "Cystic Bronchiectases"[Title/Abstract]) AND "ischemic attack, transient"[MeSH Terms]) OR "Brain TIA"[Title/Abstract] OR "tia brain"[Title/Abstract] OR "TIA"[Title/Abstract] OR "Transient Ischemic Attack"[Title/Abstract] OR "attacks transient ischemic"[Title/Abstract] OR "attack transient ischemic"[Title/Abstract] OR "ischemic attacks transient"[Title/Abstract] OR "Transient Ischemic Attacks"[Title/Abstract] OR "cerebral ischemia transient"[Title/Abstract] OR "Transient Cerebral Ischemia"[Title/Abstract] OR "Transient Cerebral Ischemias"[Title/Abstract] OR "transient ischemic attack brainstem"[Title/Abstract] OR "Transient Brainstem Ischemia"[Title/Abstract] OR "Brainstem Transient Ischemic Attack"[Title/Abstract] OR "Crescendo Transient Ischemic Attacks"[Title/Abstract] OR "Posterior Circulation Transient Ischemic Attack"[Title/Abstract] OR "Anterior Circulation Transient Ischemic Attack"[Title/Abstract] OR "Peripheral Nervous System Diseases"[MeSH Terms] OR "Peripheral Nervous System Disorders"[Title/Abstract] OR "Peripheral Neuropathies"[Title/Abstract] OR "neuropathy peripheral"[Title/Abstract] OR "Peripheral Neuropathy"[Title/Abstract] OR "Peripheral Nervous System Disease"[Title/Abstract] OR "PNS Diseases"[Title/Abstract] OR "PNS Disease"[Title/Abstract] OR "Peripheral Nerve Diseases"[Title/Abstract] OR "nerve disease peripheral"[Title/Abstract] OR "Peripheral Nerve Disease"[Title/Abstract] OR "Chronic Pain"[MeSH Terms] OR "pain chronic"[Title/Abstract] OR "Widespread Chronic Pain"[Title/Abstract] OR "chronic pain widespread"[Title/Abstract] OR "Chronic Primary Pain"[Title/Abstract] OR "pain chronic primary"[Title/Abstract] OR "primary pain chronic"[Title/Abstract] OR "Chronic Secondary Pain"[Title/Abstract] OR "pain chronic secondary"[Title/Abstract] OR "secondary pain chronic"[Title/Abstract]) AND "Neoplasms"[MeSH Terms]) OR "Tumors"[Title/Abstract] OR "Neoplasia"[Title/Abstract] OR "Neoplasias"[Title/Abstract] OR "Neoplasm"[Title/Abstract] OR "Tumor"[Title/Abstract] OR "Cancer"[Title/Abstract] OR "Cancers"[Title/Abstract] OR "Malignant Neoplasm"[Title/Abstract] OR "Malignancy"[Title/Abstract] OR "Malignancies"[Title/Abstract] OR "Malignant Neoplasms"[Title/Abstract] OR "neoplasm malignant"[Title/Abstract] OR "neoplasms malignant"[Title/Abstract] OR "Benign Neoplasms"[Title/Abstract] OR "neoplasms benign"[Title/Abstract] OR "neoplasm benign"[Title/Abstract] OR "Benign Neoplasm"[Title/Abstract]) AND "Bone Neoplasms"[MeSH Terms]) OR "Bone Neoplasm"[Title/Abstract] OR "neoplasm bone"[Title/Abstract] OR "neoplasms bone"[Title/Abstract] OR "Bone Cancer"[Title/Abstract] OR "Cancer of Bone"[Title/Abstract] OR "Cancer of the Bone"[Title/Abstract] OR "Melanoma"[MeSH Terms] OR "Melanomas"[Title/Abstract] OR "Malignant Melanoma"[Title/Abstract] OR "Malignant Melanomas"[Title/Abstract] OR "melanoma malignant"[Title/Abstract] OR "melanomas malignant"[Title/Abstract]) AND "Dementia"[MeSH Terms]) OR "Dementias"[Title/Abstract] OR "Amentia"[Title/Abstract] OR "Amentias"[Title/Abstract] OR "Familial Dementia"[Title/Abstract] OR "dementia familial"[Title/Abstract] OR "dementias familial"[Title/Abstract] OR "Familial Dementias"[Title/Abstract] OR "Schizophrenia"[MeSH Terms] OR "schizophrenia*"[Title/Abstract] OR "Dementia Praecox"[Title/Abstract] OR "Schizophrenic Disorders"[Title/Abstract] OR "disorder schizophrenic"[Title/Abstract] OR "disorders schizophrenic"[Title/Abstract] OR "Schizophrenic Disorder"[Title/Abstract] OR "Depression"[MeSH Terms] OR "Depressive Symptoms"[Title/Abstract] OR "Depressive Symptom"[Title/Abstract] OR "symptom depressive"[Title/Abstract] OR "Emotional Depression"[Title/Abstract] OR "depression emotional"[Title/Abstract] OR "Depressive Disorder"[MeSH Terms] OR "Depressive Disorders"[Title/Abstract] OR "disorder depressive"[Title/Abstract] OR "disorders depressive"[Title/Abstract] OR "neurosis depressive"[Title/Abstract] OR "Depressive Neuroses"[Title/Abstract] OR "Depressive Neurosis"[Title/Abstract] OR "neuroses depressive"[Title/Abstract] OR "depression endogenous"[Title/Abstract] OR "depressions endogenous"[Title/Abstract] OR "Endogenous Depression"[Title/Abstract] OR "Endogenous Depressions"[Title/Abstract] OR "melancholia*"[Title/Abstract] OR "Unipolar Depression"[Title/Abstract] OR "depression unipolar"[Title/Abstract] OR "depressions unipolar"[Title/Abstract] OR "Unipolar Depressions"[Title/Abstract] OR "Depressive Syndrome"[Title/Abstract] OR "Depressive Syndromes"[Title/Abstract] OR "syndrome depressive"[Title/Abstract] OR "syndromes depressive"[Title/Abstract] OR "depression neurotic"[Title/Abstract] OR "Neurotic Depression"[Title/Abstract] OR "Neurotic Depressions"[Title/Abstract] OR "Anxiety"[MeSH Terms] OR "Angst"[Title/Abstract] OR "Nervousness"[Title/Abstract] OR "Hypervigilance"[Title/Abstract] OR "Social Anxiety"[Title/Abstract] OR "anxiety social"[Title/Abstract] OR "Social Anxieties"[Title/Abstract] OR "Anxiousness"[Title/Abstract] OR "Bipolar Disorder"[MeSH Terms] OR "Bipolar Disorders"[Title/Abstract] OR "disorder bipolar"[Title/Abstract] OR "Bipolar Mood Disorder"[Title/Abstract] OR "Bipolar Mood Disorders"[Title/Abstract] OR "disorder bipolar mood"[Title/Abstract] OR "mood disorder bipolar"[Title/Abstract] OR "affective psychosis bipolar"[Title/Abstract] OR "Bipolar Affective Psychosis"[Title/Abstract] OR "psychoses bipolar affective"[Title/Abstract] OR "psychosis bipolar affective"[Title/Abstract] OR "psychoses manic depressive"[Title/Abstract] OR "psychoses manic depressive"[Title/Abstract] OR "manic depressive psychosis"[Title/Abstract] OR "manic depressive psychosis"[Title/Abstract] OR "psychosis manic depressive"[Title/Abstract] OR "psychosis manic depressive"[Title/Abstract] OR "depression bipolar"[Title/Abstract] OR "Bipolar Depression"[Title/Abstract] OR "Manic Disorder"[Title/Abstract] OR "disorder manic"[Title/Abstract] OR "Manic Disorders"[Title/Abstract] OR "Manic Depression"[Title/Abstract] OR "depression manic"[Title/Abstract] OR "depressions manic"[Title/Abstract] OR "Bipolar Disorder Type 1"[Title/Abstract] OR "Type 1 Bipolar Disorder"[Title/Abstract] OR "Bipolar Disorder Type 2"[Title/Abstract] OR "Type 2 Bipolar Disorder"[Title/Abstract] OR "Drug Misuse"[MeSH Terms] OR "Drug Misuses"[Title/Abstract] OR "misuse drug"[Title/Abstract] OR "non prescription medicine misuse"[Title/Abstract] OR "non prescription medicine misuse"[Title/Abstract] OR "Nonprescription Drug Misuse"[Title/Abstract] OR "over the counter drug misuse"[Title/Abstract] OR "misuse over the counter drug"[Title/Abstract] OR "over the counter drug misuse"[Title/Abstract] OR "Feeding and Eating Disorders"[MeSH Terms] OR "Eating and Feeding Disorders"[Title/Abstract] OR "Appetite Disorders"[Title/Abstract] OR "Appetite Disorder"[Title/Abstract] OR "Eating Disorders"[Title/Abstract] OR "disorder eating"[Title/Abstract] OR "disorders eating"[Title/Abstract] OR "Eating Disorder"[Title/Abstract] OR "Feeding Disorders"[Title/Abstract] OR "disorder feeding"[Title/Abstract] OR "disorders feeding"[Title/Abstract] OR "Feeding Disorder"[Title/Abstract] OR "Autistic Disorder"[MeSH Terms] OR "disorder autistic"[Title/Abstract] OR "disorders autistic"[Title/Abstract] OR "Autism"[Title/Abstract] OR "autism early infantile"[Title/Abstract] OR "Early Infantile Autism"[Title/Abstract] OR "infantile autism early"[Title/Abstract] OR "autism infantile"[Title/Abstract] OR "Infantile Autism"[Title/Abstract] OR "Kanner's Syndrome"[Title/Abstract] OR "Kanners Syndrome"[Title/Abstract] OR "Kanner Syndrome"[Title/Abstract] OR "stress disorders, post traumatic"[MeSH Terms] OR "post traumatic stress disorder"[Title/Abstract] OR "stress disorder post traumatic"[Title/Abstract] OR "post traumatic stress disorder"[Title/Abstract] OR "neuroses post traumatic"[Title/Abstract] OR "neuroses post traumatic"[Title/Abstract] OR "Post-Traumatic Neuroses"[Title/Abstract] OR "PTSD"[Title/Abstract] OR "stress disorder post traumatic"[Title/Abstract] OR "post traumatic stress disorders"[Title/Abstract] OR "post traumatic stress disorders"[Title/Abstract] OR "Posttraumatic Stress Disorders"[Title/Abstract] OR "Posttraumatic Stress Disorder"[Title/Abstract] OR "stress disorder posttraumatic"[Title/Abstract] OR "stress disorders posttraumatic"[Title/Abstract] OR "Posttraumatic Neuroses"[Title/Abstract] OR "acute post traumatic stress disorder"[Title/Abstract] OR "acute post traumatic stress disorder"[Title/Abstract] OR "chronic post traumatic stress disorder"[Title/Abstract] OR "chronic post traumatic stress disorder"[Title/Abstract] OR "delayed onset post traumatic stress disorder"[Title/Abstract] OR "delayed onset post traumatic stress disorder"[Title/Abstract] OR "Moral Injury"[Title/Abstract] OR "injury moral"[Title/Abstract] OR "Moral Injuries"[Title/Abstract]) AND "Musculoskeletal Diseases"[MeSH Terms]) OR "Musculoskeletal Disease"[Title/Abstract] OR "orthopedic disorder*"[Title/Abstract] OR "orthopaedic disorder*"[Title/Abstract] OR "Connective Tissue Diseases"[MeSH Terms] OR "Connective Tissue Disease"[Title/Abstract] OR "disease connective tissue"[Title/Abstract] OR "diseases connective tissue"[Title/Abstract] OR "Arthritis"[MeSH Terms] OR "Arthritides"[Title/Abstract] OR "Polyarthritis"[Title/Abstract] OR "Polyarthritides"[Title/Abstract] OR "Oligoarthritis"[Title/Abstract] OR "Oligoarthritides"[Title/Abstract] OR "Osteoarthritis"[MeSH Terms] OR "Osteoarthritides"[Title/Abstract] OR "arthritis degenerative"[Title/Abstract] OR "Degenerative Arthritides"[Title/Abstract] OR "Degenerative Arthritis"[Title/Abstract] OR "Osteoarthrosis"[Title/Abstract] OR "Osteoarthroses"[Title/Abstract] OR "Osteoarthrosis Deformans"[Title/Abstract] OR "Arthrosis"[Title/Abstract] OR "Arthroses"[Title/Abstract] OR "Osteoporosis"[MeSH Terms] OR "Osteoporoses"[Title/Abstract] OR "Osteoporosis"[Title/Abstract] OR "osteoporosis age related"[Title/Abstract] OR "osteoporosis age related"[Title/Abstract] OR "age related osteoporosis"[Title/Abstract] OR "Age-Related Osteoporoses"[Title/Abstract] OR "age related osteoporosis"[Title/Abstract] OR "bone loss age related"[Title/Abstract] OR "Age-Related Bone Loss"[Title/Abstract] OR "Age-Related Bone Losses"[Title/Abstract] OR "bone loss age related"[Title/Abstract] OR "osteoporosis senile"[Title/Abstract] OR "Senile Osteoporosis"[Title/Abstract] OR "osteoporosis post traumatic"[Title/Abstract] OR "osteoporosis post traumatic"[Title/Abstract] OR "Post-Traumatic Osteoporosis"[Title/Abstract] OR "Gout"[MeSH Terms] OR "gout*"[Title/Abstract] OR "chronic digestive disease"[Title/Abstract] OR "chronic gastrointestinal disease"[Title/Abstract] OR "chronic GI disorders"[Title/Abstract] OR "chronic intestinal diseases"[Title/Abstract] OR "gastroesophageal reflux disease"[Title/Abstract] OR "GERD"[Title/Abstract] OR "chronic irritable bowel syndrome"[Title/Abstract] OR "Crohn's disease"[Title/Abstract] OR "chronic ulcerative colitis"[Title/Abstract] OR "Celiac Disease"[Title/Abstract] OR "chronic peptic ulcer"[Title/Abstract] OR "chronic diverticulitis"[Title/Abstract] OR "chronic gallbladder disease"[Title/Abstract] OR "chronic liver disease"[Title/Abstract] OR "chronic pancreatitis"[Title/Abstract] OR "chronic abdominal pain"[Title/Abstract] OR "chronic diarrhea"[Title/Abstract] OR "chronic constipation"[Title/Abstract] OR "chronic dyspepsia"[Title/Abstract] OR "chronic nausea and vomiting"[Title/Abstract] OR "chronic dysbiosis"[Title/Abstract] OR "chronic motility disorders"[Title/Abstract] OR "chronic malabsorption syndromes"[Title/Abstract] OR "Gastroesophageal Reflux"[MeSH Terms] OR "Irritable Bowel Syndrome"[MeSH Terms] OR "Crohn Disease"[MeSH Terms] OR "Celiac Disease"[MeSH Terms] OR "End Stage Liver Disease"[MeSH Terms] OR "pancreatitis, chronic"[MeSH Terms] OR "Ciliary Motility Disorders"[MeSH Terms] OR "chronic kidney disease"[Title/Abstract] OR "end-stage kidney disease"[Title/Abstract] OR "end-stage renal disease"[Title/Abstract] OR "chronic renal failure"[Title/Abstract] OR "Renal Insufficiency"[Title/Abstract] OR "dialysis-dependent kidney disease"[Title/Abstract] OR "Endometriosis"[Title/Abstract] OR "deep infiltrating endometriosis"[Title/Abstract] OR "chronic pelvic pain"[Title/Abstract] OR "endometriosis-associated infertility"[Title/Abstract] OR "chronic urinary tract infection"[Title/Abstract] OR "recurrent urinary tract infection"[Title/Abstract] OR "interstitial cystitis"[Title/Abstract] OR "bladder pain syndrome"[Title/Abstract] OR "kidney failure, chronic"[MeSH Terms] OR "Renal Insufficiency"[MeSH Terms] OR "Endometriosis"[MeSH Terms] OR "cystitis, interstitial"[MeSH Terms] OR "Thalassemia"[Title/Abstract] OR "sickle cell disease"[Title/Abstract] OR "Polycythemia Vera"[Title/Abstract] OR "essential thrombocythemia"[Title/Abstract] OR "Myelodysplastic Syndromes"[Title/Abstract] OR "chronic myeloid leukemia"[Title/Abstract] OR "chronic lymphocytic leukemia"[Title/Abstract] OR "Lymphoma"[Title/Abstract] OR "Hodgkin lymphoma"[Title/Abstract] OR "non-Hodgkin lymphoma"[Title/Abstract] OR "Multiple Myeloma"[Title/Abstract] OR "Hemochromatosis"[Title/Abstract] OR "paroxysmal nocturnal hemoglobinuria"[Title/Abstract] OR "beta thalassemia"[Title/Abstract] OR "alpha thalassemia"[Title/Abstract] OR "Hemoglobinopathies"[Title/Abstract] OR "plasma cell dyscrasias"[Title/Abstract] OR "Thalassemia"[MeSH Terms] OR "anemia, sickle cell"[MeSH Terms] OR "Polycythemia Vera"[MeSH Terms] OR "thrombocythemia, essential"[MeSH Terms] OR "Myelodysplastic Syndromes"[MeSH Terms] OR "Leukemia"[MeSH Terms] OR "Lymphoma"[MeSH Terms] OR "Hodgkin Disease"[MeSH Terms] OR "Multiple Myeloma"[MeSH Terms] OR "Hemochromatosis"[MeSH Terms] OR "hemoglobinuria, paroxysmal"[MeSH Terms] OR "beta thalassemia"[MeSH Terms] OR "alpha thalassemia"[MeSH Terms] OR "Hemoglobinopathies"[MeSH Terms] OR "Paraproteinemias"[MeSH Terms] OR "irreversible vision loss"[Title/Abstract] OR "Blindness"[Title/Abstract] OR "age-related macular degeneration"[Title/Abstract] OR "ARMD"[Title/Abstract] OR "Retinitis Pigmentosa"[Title/Abstract] OR "Glaucoma"[Title/Abstract] OR "Diabetic Retinopathy"[Title/Abstract] OR "Cataract"[Title/Abstract] OR "optic neuropathy"[Title/Abstract] OR "Uveitis"[Title/Abstract] OR "corneal scarring"[Title/Abstract] OR "albinism"[Title/Abstract] OR "leber congenital amaurosis"[Title/Abstract] OR "Optic Atrophy"[Title/Abstract] OR "Retinal Detachment"[Title/Abstract] OR "macular hole"[Title/Abstract] OR "dry eye"[Title/Abstract] OR "Aniridia"[Title/Abstract] OR "Choroideremia"[Title/Abstract] OR "Stargardt Disease"[Title/Abstract] OR "fuchs dystrophy"[Title/Abstract] OR "cone-rod dystrophy"[Title/Abstract] OR "nystagmus"[Title/Abstract] OR "Amblyopia"[Title/Abstract] OR "visual field loss"[Title/Abstract] OR "Blindness"[MeSH Terms] OR "Retinitis Pigmentosa"[MeSH Terms] OR "Glaucoma"[MeSH Terms] OR "Diabetic Retinopathy"[MeSH Terms] OR "Cataract"[MeSH Terms] OR "optic neuropathy, ischemic"[MeSH Terms] OR "Uveitis"[MeSH Terms] OR "Corneal Injuries"[MeSH Terms] OR "albinism, ocular"[MeSH Terms] OR "leber congenital amaurosis"[MeSH Terms] OR "Optic Atrophy"[MeSH Terms] OR "Retinal Detachment"[MeSH Terms] OR "Retinal Diseases"[MeSH Terms] OR "Dry Eye Syndromes"[MeSH Terms] OR "Aniridia"[MeSH Terms] OR "Choroideremia"[MeSH Terms] OR "Stargardt Disease"[MeSH Terms] OR "Cone-Rod Dystrophies"[MeSH Terms] OR "nystagmus, congenital"[MeSH Terms] OR "Amblyopia"[MeSH Terms]) AND "Meniere's disease"[Title/Abstract]) OR "sensorineural hearing loss"[Title/Abstract] OR "congenital deafness"[Title/Abstract] OR "age-related hearing loss"[Title/Abstract] OR "Presbycusis"[Title/Abstract] OR "noise-induced hearing loss"[Title/Abstract] OR "Ototoxicity"[Title/Abstract] OR "Meniere's disease"[Title/Abstract] OR "genetic hearing loss"[Title/Abstract] OR "autoimmune inner ear disease"[Title/Abstract] OR "auditory neuropathy spectrum disorder"[Title/Abstract] OR "Usher syndrome"[Title/Abstract] OR "waardenburg syndrome"[Title/Abstract] OR "Pendred syndrome"[Title/Abstract] OR "Alport syndrome"[Title/Abstract] OR "labyrinthitis"[Title/Abstract] OR "viral cochleitis"[Title/Abstract] OR "acoustic neuroma"[Title/Abstract] OR "bilateral vestibular schwannoma"[Title/Abstract] OR "Cholesteatoma"[Title/Abstract] OR "perilymph fistula"[Title/Abstract] OR "trauma-induced hearing loss"[Title/Abstract] OR "permanent hearing loss"[Title/Abstract] OR "irreversible hearing loss"[Title/Abstract] OR "sensorineural hearing loss"[Title/Abstract] OR "chronic hearing impairment"[Title/Abstract] OR "sensorineural deafness"[Title/Abstract] OR "permanent deafness"[Title/Abstract] OR "hearing loss rehabilitation"[Title/Abstract] OR "hearing loss management"[Title/Abstract] OR "congenital hearing impairment"[Title/Abstract] OR "Deafness"[MeSH Terms] OR "hearing loss, sensorineural"[MeSH Terms] OR "deafness congenital with inner ear agenesis microtia and microdontia"[Supplementary Concept] OR "Presbycusis"[MeSH Terms] OR "hearing loss, noise induced"[MeSH Terms] OR "Ototoxicity"[MeSH Terms] OR "Meniere Disease"[MeSH Terms] OR "Usher Syndromes"[MeSH Terms] OR "waardenburg syndrome"[MeSH Terms] OR "Pendred syndrome"[Supplementary Concept] OR "neuroma, acoustic"[MeSH Terms] OR "Cholesteatoma"[MeSH Terms] OR "HIV"[Title/Abstract] OR "Human Immunodeficiency Virus"[Title/Abstract] OR "AIDS"[Title/Abstract] OR "Acquired Immunodeficiency Syndrome"[Title/Abstract] OR "HIV Infection"[Title/Abstract] OR "HIV Disease"[Title/Abstract] OR "AIDS Virus"[Title/Abstract] OR "HIV Positive"[Title/Abstract] OR "HIV/AIDS"[Title/Abstract] OR "HIV/AIDS Infections"[Title/Abstract] OR "HIV"[MeSH Terms] OR "Acquired Immunodeficiency Syndrome"[MeSH Terms] OR "Tuberculosis"[MeSH Terms] OR "Tuberculosis"[Title/Abstract] OR "TB"[Title/Abstract] OR "chronic Lyme disease"[Title/Abstract] OR "post-treatment Lyme disease syndrome"[Title/Abstract] OR "post-Lyme disease"[Title/Abstract] OR "chronic symptoms of Lyme disease"[Title/Abstract] OR "persistent Lyme disease"[Title/Abstract] OR "Post-Lyme Disease Syndrome"[MeSH Terms] OR "post-acute COVID-19"[Title/Abstract] OR "long COVID"[Title/Abstract] OR "post-COVID syndrome"[Title/Abstract] OR "chronic COVID-19 symptoms"[Title/Abstract] OR "COVID-19 long-term effects"[Title/Abstract] OR "post-viral fatigue syndrome"[Title/Abstract] OR "persistent COVID symptoms"[Title/Abstract] OR "post-acute sequelae of SARS-CoV-2 infection"[Title/Abstract] OR "post-COVID-19 rehabilitation"[Title/Abstract] OR "Post-Acute COVID-19 Syndrome"[MeSH Terms] OR "COVID-19 post-intensive care syndrome"[Supplementary Concept] OR "Congenital Disorders"[Title/Abstract] OR "Congenital Anomalies"[Title/Abstract] OR "Congenital Malformations"[Title/Abstract] OR "Birth Defects"[Title/Abstract] OR "Prenatal Abnormalities"[Title/Abstract] OR "Genetic Birth Defects"[Title/Abstract] OR "Inborn Errors of Metabolism"[Title/Abstract] OR "Congenital Syndromes"[Title/Abstract] OR "Congenital Heart Defects"[Title/Abstract] OR "Neonatal Disorders"[Title/Abstract] OR "Congenital Brain Abnormalities"[Title/Abstract] OR "Congenital Skeletal Abnormalities"[Title/Abstract] OR "Neonatal Genetic Disorders"[Title/Abstract] OR "Prenatal Diagnoses"[Title/Abstract] OR "Embryonic Development Disorders"[Title/Abstract] OR "Chromosomal Disorders"[Title/Abstract] OR "Chromosomal Abnormalities"[Title/Abstract] OR "Chromosome Disorders"[Title/Abstract] OR "Chromosome Aberrations"[Title/Abstract] OR "Down Syndrome"[Title/Abstract] OR "Klinefelter Syndrome"[Title/Abstract] OR "Turner Syndrome"[Title/Abstract] OR "Patau Syndrome"[Title/Abstract] OR "Edward Syndrome"[Title/Abstract] OR "Trisomy Disorders"[Title/Abstract] OR "Structural Chromosome Abnormalities"[Title/Abstract] OR "Balanced Chromosome Translocation"[Title/Abstract] OR "Unbalanced Chromosome Aberrations"[Title/Abstract] OR "Cytogenetic Abnormalities"[Title/Abstract] OR "Autosomal Dominant Inheritance"[Title/Abstract] OR "Autosomal Recessive Disorders"[Title/Abstract] OR "X-linked Diseases"[Title/Abstract] OR "Mitochondrial Genetic Disorders"[Title/Abstract] OR "Genetic Abnormalities"[Title/Abstract] OR "Inherited Disorders"[Title/Abstract] OR "Genomic Disorders"[Title/Abstract] OR "Genetic Counseling"[Title/Abstract] OR "congenital, hereditary, and neonatal diseases and abnormalities"[MeSH Terms])) AND ("community based participatory research"[Title/Abstract] OR "Participatory Research Community-Based"[Title/Abstract] OR "consumer driven community based research"[Title/Abstract] OR "consumer driven community based research"[Title/Abstract] OR "consumer driven community based research"[Title/Abstract] OR "Participation Community"[Title/Abstract] OR "community involv*"[Title/Abstract] OR "Involvement Community"[Title/Abstract] OR "community action*"[Title/Abstract] OR "Action Community"[Title/Abstract] OR "Consumer Participation"[Title/Abstract] OR "Participation Consumer"[Title/Abstract] OR "consumer involv*"[Title/Abstract] OR "Involvement Consumer"[Title/Abstract] OR "Public Participation"[Title/Abstract] OR "Participation Public"[Title/Abstract] OR "stakeholder role*"[Title/Abstract] OR "stakeholder engagement*"[Title/Abstract] OR "Engagement Stakeholder"[Title/Abstract] OR "participa*"[Title/Abstract] OR "Participation"[Title/Abstract] OR "Participation Patient"[Title/Abstract] OR "Patient Involvement"[Title/Abstract] OR "Involvement Patient"[Title/Abstract] OR "Patient Participation Rates"[Title/Abstract] OR "Participation Rates Patient"[Title/Abstract] OR "Patient Participation Rate"[Title/Abstract] OR "Patient Engagement"[Title/Abstract] OR "Engagement Patient"[Title/Abstract] OR "Patient Activation"[Title/Abstract] OR "Activation Patient"[Title/Abstract] OR "Patient Empowerment"[Title/Abstract] OR "Empowerment Patient"[Title/Abstract] OR "Participant involvement"[Title/Abstract] OR "Participation Community"[Title/Abstract] OR "community involvement*"[Title/Abstract] OR "involvement* community"[Title/Abstract] OR "community action*"[Title/Abstract] OR "Action Community"[Title/Abstract] OR "Actions Community"[Title/Abstract] OR "Consumer Participation"[Title/Abstract] OR "Participation Consumer"[Title/Abstract] OR "consumer involv*"[Title/Abstract] OR "involv* consumer"[Title/Abstract] OR "Public Participation"[Title/Abstract] OR "Participation Public"[Title/Abstract] OR "participatory action research"[Title/Abstract] OR "Community engagement"[Title/Abstract] OR "community involve*"[Title/Abstract] OR "Community Participation"[Title/Abstract] OR "Patient Participation"[Title/Abstract] OR "Stakeholder Participation"[Title/Abstract] OR "community based participatory research"[MeSH Terms] OR "Community Participation"[MeSH Terms] OR "Patient Participation"[MeSH Terms] OR "Stakeholder Participation"[MeSH Terms]) AND ("theory of change"[Title/Abstract] OR "participatory model"[Title/Abstract] OR "shared decision-making model"[Title/Abstract] OR "engagement framework"[Title/Abstract] OR "involvement framework"[Title/Abstract] OR "patient-centered care model"[Title/Abstract] OR "self-management framework"[Title/Abstract] OR "chronic care model"[Title/Abstract] OR "community engagement model"[Title/Abstract] OR "co-production model"[Title/Abstract] OR "co-design framework"[Title/Abstract] OR "social-ecological model"[Title/Abstract] OR "health belief model"[Title/Abstract] OR "behavioral change theory"[Title/Abstract] OR "transtheoretical model"[Title/Abstract] OR "collaborative care model"[Title/Abstract] OR "empowerment theory"[Title/Abstract] OR "person-centered care framework"[Title/Abstract])
