# Supplementary material for: Towards meaningful engagement: theoretical approaches guiding the participation of people living with multiple long-term conditions in health research – a scoping review
Source: BMJ Public Health. 2026 Sep 25;4(3):e004602. doi: 10.1136/bmjph-2025-004602 (PMC13630058; doi:10.1136/bmjph-2025-004602)
Supplement: Supplementary file 3 [file bmjph-4-3-s003.docx]

**Supplementary File 3**

| **Data charting form** | |
| --- | --- |
| **Data Item** | **Description** |
| Author(s) | List of authors involved in the study. |
| Title | Title of the study. |
| Year of Publication | The year study was published. |
| Study Aim and Objectives | The primary aim and specific objectives of the study. |
| Study Setting (Geographical/Contextual Details) | Context of the study including geographical location (e.g., LMICs, HICs) or specific contextual information. |
| Study Population | The population under study (e.g., people living with MLTCs, community members). |
| Participant Demographics | Demographic characteristics (e.g., age, gender, socio-economic status, ethnicity). |
| Baseline Characteristics | Key characteristics of participants before the intervention (e.g., health conditions, medical history). |
| CEI Models/Frameworks/Theories Used | Specific community engagement and involvement models, frameworks, or theories discussed in the study. |
| Constructs/Concepts/Dimensions of the Models/Frameworks/Theories | Key concepts, constructs, or dimensions of the CEI models/frameworks/theories used in the study. |
| Methods of Engagement/Involvement | Description of the methods or strategies employed for engagement or involvement of people with MLTCs. |
| Aspects of Community Engagement and Involvement | E.g: Education & Awareness, Community-based care delivery, Participation in decision-making, Behaviour changes etc. |
| Level of implication/ implementation of the Models/Frameworks/Theories | E.g. health system & individual level etc. |
| Number and Conditions (Diseases) Covered | The number of conditions included in the study and the specific diseases or conditions addressed. |
| Outcomes | Key outcomes or results measured (e.g., health improvements, engagement levels, satisfaction). |
| Result | Theories/Frameworks/Models of applicability |
| Times of Measurement | Timepoints at which measurements or outcomes were assessed (e.g., baseline, post-intervention, follow-up). |
| Data Item | Description |
