# Supplementary material for: Towards meaningful engagement: theoretical approaches guiding the participation of people living with multiple long-term conditions in health research – a scoping review
Source: BMJ Public Health. 2026 Sep 25;4(3):e004602. doi: 10.1136/bmjph-2025-004602 (PMC13630058; doi:10.1136/bmjph-2025-004602)
Supplement: Supplementary file 4 [file bmjph-4-3-s004.docx]

**Supplementary File 4**

| **TFMs, and engagement strategies for people with MLTC** | | | | |
| --- | --- | --- | --- | --- |
| **Study details** | **Objectives** | **Level of implication and aspects of CEI** | **Result (Applicability of the TFMs)** | **Factors associated with the TFMs** |
| **TFM: Chronic Care Model (CCM), (n=2)** | | | | |
| **Citation:** (Towfighi et al., 2021a) | Determine if a CCM, community health worker (CHW), advanced practice clinician, and physician team intervention improves risk factor control after stroke in a safety-net setting. | **Level of implication: Health system**: Safety-net hospitals and clinics; **Provider:** Advanced practice clinicians, CHWs, physicians; **Patient:** Adults with recent stroke or transient ischemic attack (TIA). | - **Health outcomes:** No significant difference in systolic blood pressure (SBP) control at 12 months between groups; 45% of intervention and 44% of usual care participants achieved BP ≤130mmHg. - **Socio-behavioural:** Intervention participants had a 15% higher rate of self-reported reduction in salt intake compared to usual care (p=0.004). - **System level:** CCM is feasible to deliver in safety-net settings but showed low full participation rates; usual care improvements and other structural factors likely influenced results. | **Positive:** Intervention helped improve lifestyle behaviours. |
| **Country**: United States of America (USA). |  | **Aspects of CEI:**   - **Health Education**: Provided culturally tailored materials and Chronic Disease Self-Management Program workshops to improve stroke knowledge. - **Community Participation**: Engaged patients through clinic visits, home visits by CHWs, and group workshops. - **Collaborative Support**: A multidisciplinary team: CHWs, clinicians, and physicians coordinated care and supported patients. |  | **Negative:** No significant effect on systolic blood pressure (SBP) control compared to usual care. |
| **Conditions:** Hypertension and Stroke. |  |  |  | **Neutral:** N/A. |
| **Citation:** (Lopez et al., 2024) | Examine the Salud y Vida program's effect on reducing Blood pressure (BP) over time between groups with higher and lower engagement. | **Level of implementation: Health system:** Expanded CCM with CHWs; **Provider**: CHWs and diabetes educators; **Patient**: Hispanic adults with poorly controlled type 2 diabetes and hypertension. | - **Health outcomes:** SBP reduced by 6.5mmHg and Diastolic blood pressure (DBP) by 4.0mmHg over 3 months; greater reductions in the higher engagement group (SBP −7.7mmHg *vs.* −5.6mmHg). - **Socio-behavioural:** 21–23% had baseline depression; higher engagement was more common among Spanish speakers (75% vs. 69%), but linked to lower employment and slightly lower education levels. - **System level:** N/A. | **Positive:** Intervention improved BP outcomes with higher engagement. |
| **Country**: USA. |  | **Aspects of CEI:**   - **Health Education**: Delivered diabetes and BP self-management classes. - **Community Participation**: Engaged participants through home visits, goal-setting, and regular follow-up. - **Collaborative Support**: CHWs worked alongside health educators and clinicians to coordinate and support care. |  | **Negative:** BP reduction did not reach clinically significant thresholds. |
| **Conditions:** Hypertension and Type 2 Diabetes. |  |  |  | **Neutral:** N/A. |
| **TFM: Collaborative Care Model (CoCM), (n = 14)** | | | | |
| **Citation:** (Coventry et al., 2015) | Test the effectiveness of an integrated CoCM for people with depression and long term  physical conditions. | **Level of implication: Health system:** Primary care general practices; **Provider:** Psychological practitioners and practice nurses; **Patient**: Adults with depression and diabetes and/or coronary heart disease with multimorbidity. | - **Health outcomes:** Collaborative care reduced depression by 0.23 points on SCL-D13 (effect size −0.30) and anxiety by 1.45 points on Generalized Anxiety Disorder 7-item (GAD-7) (effect size −0.28) at 4 months. - **Socio-behavioural:** Patients showed improved self-management skills with improvements in skill acquisition (effect size 0.25), constructive attitudes (0.25), self-monitoring (0.36), and rated care as more patient-centered (Patient Assessment of Chronic Illness Care - total score effect size 0.39). - **Health system level:** Collaborative care delivered by trained psychological wellbeing practitioners integrated with nurses in primary care can be implemented with minimal changes to usual care. | **Positive:** Modest depression and anxiety improvement. |
| **Country:** England. |  | **Aspects of CEI:**   - **Health Education:** Delivered brief psychological therapies and training to improve depression and self-management in patients. - **Community Participation**: Engaged patients actively through psychological sessions, joint meetings with nurses, and personalized goal-setting. - **Collaborative Support:** Multidisciplinary team including psychological wellbeing practitioners and primary care nurses to integrate mental and physical health care. |  | **Negative:** Modest effect size; no significant improvement in disability or quality of life. |
| **Conditions:** Depression, Diabetes and Cardiovascular Disease. |  |  |  | **Neutral:** N/A. |
| **Study Citation****:** (Beck et al., 2018) | Describe commonalities and variations in multilevel measures associated with implementation of Care of Mental, Physical, and Substance-Use Syndromes (COMPASS), a large-scale collaborative care intervention for depression, diabetes, and cardiovascular disease. | **Level of implication:** **Health system**: Health care systems across 8 states; **Provider**: Care managers (nurses, social workers, psychologists) with physician and psychiatrist consultants; **Patient**: Adults with depression and poorly controlled diabetes and/or cardiovascular disease. | - **Health outcomes**: Improvements in depression; longer patient enrolment (1–26 months) associated with better outcomes. - **Socio-behavioural**: >50% of patients had high social needs (e.g., transportation, food, housing), requiring care managers to spend substantial additional time arranging support; motivational interviewing helped engage patients. - **Health system level:** Implementation was influenced by organizational culture, primary care providers engagement, care manager training, registry use was generally low, and funding and staff turnover challenges impacted sustainability. | **Positive**: Improved team collaboration and tailored patient management. |
| **Country:** USA. |  | **Aspects of CEI:**   - **Health Education:** Provided evidence-based information and behavioural strategies to help patients understand and manage depression and chronic diseases. - **Community Participation:** Engaged patients and caregivers through regular goal-setting, follow-ups, and involvement in care planning. - **Collaborative Support:** Coordinated care via a multidisciplinary team—physicians, psychiatrists, care managers—working together to address medical, behavioural, and social needs. |  | **Negative:** Implementation challenges including provider engagement variability and reimbursement limitations. |
| **Conditions:** Depression, Diabetes and Cardiovascular Disease. |  |  |  | **Neutral:** N/A. |
| **Citation:** (Fuller et al., 2019) | Explore patient experiences and attitudes about services received through the CoCM, including measurement-based care using the Patient Health Questionnaire-9 (PHQ-9). | **Level of implication: Health system**: Safety-net HIV primary care clinic; **Provider**: HIV care providers, social worker care coordinators, consulting psychiatrist; **Patient**: Adults with HIV and depression. | - **Health outcomes:** N/A. - **Socio-behavioural:** CoCM encouraged new coping strategies and mental health therapy initiation among patients. - **System (health system) level:** Collaborative care integrated mental health into HIV care with minimal disruption, improving coordination and addressing workforce shortages. | **Positive:** CoCM for HIV and depression (enhanced patient trust, routine depression screening with PHQ-9, supported self-management). |
| **Country:** USA. |  | **Aspects of CEI:**   - **Health Education:** Provided depression screening and self-management support using PHQ-9 and tailored mental health education. - **Community Participation**: Through routine depression screening, care coordination, and involvement in mental health services. - **Collaborative Support**: Multidisciplinary team of HIV providers, care coordinators, and psychiatrists to coordinate and deliver comprehensive care. |  | **Negative:** N/A. |
| **Conditions:** Human immunodeficiency virus (HIV) and Depression. |  |  |  | **Neutral:** N/A. |
| **Study Citation:** (Balasundaram et al., 2023) | Investigate differential engagement of participants in the collaborative care intervention for diabetes and depression and examine how varying levels of engagement influenced participant outcomes over a 3-year period following randomization. | **Level of implication:** **Health system**: Urban diabetes clinics; **Provider**: Care coordinators, psychiatrists, diabetologists, diabetes physicians; **Patient**: Adults with co-morbid depression and diabetes. | - **Health outcomes**: At 12 months, 83.6% of high engagers and 60% of moderate engagers improved depression plus cardio-metabolic health. - **Socio-behavioural:** High engagers had more severe symptoms but experienced more relapse (38.1%) by 36 months, indicating complex psychosocial needs. - **System (health system) level:** Collaborative care using non-physician care coordinators and specialists was feasible in diverse urban diabetes clinics. | **Positive:** Improved depression and cardio-metabolic outcomes, especially with moderate engagement. |
| Country: India. |  | **Aspects of CEI:**   - **Health Education:** Delivered culturally relevant information and coaching to improve self-management of diabetes and depression. - **Community Participation:** Engaged patients through scheduled intervention visits, goal-setting, and family involvement in treatment. - **Collaborative Support:** A multidisciplinary team: care coordinators, psychiatrists, diabetologists, and physicians to deliver integrated care. |  | **Negative:** High engagement showed greater relapse after intervention ended. |
| **Conditions:** Diabetes and Depression. |  |  |  | **Neutral**: N/A. |
| **Study Citation:** (Srinivasan, Heylen, et al., 2022) | Evaluate the effectiveness of a CoCM compared to enhanced standard treatment in improving depression outcomes among primary health care patients in rural South India who are diagnosed with co-morbid chronic medical conditions (such as diabetes or cardiovascular diseases), using a cluster randomized controlled trial design. | **Level of implication:** **Health system**: Primary Health Centre (PHCs); **Provider**: physicians, nurses (care managers), pharmacists, Accredited Social Health Activists (ASHAs); **Patient**: Adults with depression and co-morbid chronic medical conditions (hypertension, diabetes, ischemic heart disease) | - **Health outcomes:** Collaborative care reduced depression scores significantly more than enhanced standard care at 3, 6, and 12 months; at 12 months, mean PHQ-9 was 4.22 (SD 2.65) *vs.* 5.23 (SD 2.71) (p < 0.001). - **Socio-behavioural:** Collaborative care participants had greater antidepressant use (around 27% *vs*. ~1% in control) and engagement supported by ASHAs who facilitated healthy living groups and family involvement. - **System (health system) level:** CoCM delivered via trained PHC teams with weekly psychiatrist support across 24 rural PHCs; control PHCs also received basic clinician training; 6 PHCs needed replacement due to staffing issues. | **Positive**: CoCM improved depression outcomes and anti-depressant use among patients with co-morbid medical conditions. |
| **Country:** India. |  | **Aspects of CEI:**   - **Health Education:** Delivered group sessions and materials on depression, chronic disease management, and healthy living to improve participants’ knowledge and self-management skills. - **Community Participation:** Engaged participants through community-based “healthy living” groups, home visits by ASHAs, and family involvement in supporting healthy behaviours. - **Collaborative Support:** Coordinated care via a multidisciplinary team: primary care physicians, nurses, pharmacists, and ASHAs, with regular training and psychiatric consultation to integrate mental and physical health care. |  | **Negative:** N/A |
| **Conditions:** Diabetes, Hypertension, Depression, and ischemic heart disease. |  |  |  | **Neutral:** N/A |
| **Study Citation:** (Lawrence-Sidebottom et al., 2024) | 1. Characterize rates of trauma, Post-traumatic Stress Disorder (PTSD), and comorbid anxiety and depressive symptoms. 2. Determine whether trauma exposure and elevated PTSD symptoms impact the improvement of comorbid anxiety and depressive symptoms throughout participation in care.  3. Determine whether participation in a non–posttraumatic digital mental health interventions (DMHI) is linked to reductions in PTS symptoms. | **Level of implication:** **Health system**: Paediatric digital mental health platform using CoCM; **Provider**: Therapists, coaches, behavioural care managers, psychiatrists; **Patient**: Children (6-12 years) with trauma, anxiety, depression, or Attention-Deficit/Hyperactivity Disorder (ADHD) symptoms. | - **Health outcomes:** 30% youth reported trauma; 41% had elevated posttraumatic stress (PTS); 96% with elevated PTS showed significant symptom reductions; anxiety symptoms improved slower in PTS youth; depressive symptom improvements were similar. - **Socio-behavioural**: Youth with elevated PTS had higher anxiety (73%) and depression (50%) rates; more among females (56%); higher participation in anxiety and depression programs. - **System (health system) level:** Collaborative care DMHI used digital platforms with behavioural care managers, therapists, and psychiatrists; median care duration ~3 months; routine screening and symptom tracking enabled. | **Positive:** Paediatric CoCM improved anxiety, depression, and posttraumatic stress symptoms in trauma-exposed youth. |
| **Country:** USA. |  | **Aspects of CEI:**   - **Health Education**: Delivered behavioural care including cognitive behavioural therapy and caregiver training through digital sessions for anxiety and depression. - **Community Participation:** Engaged children and caregivers actively via digital coaching and therapy sessions with regular symptom assessments and individualized care plans. - **Collaborative Support:** Coordinated multidisciplinary care involving behavioural care managers, primary care providers, psychiatrists, therapists, and coaches to support whole-family mental health. |  | **Negative:** Youth with elevated posttraumatic stress symptoms showed slower anxiety symptom improvement. |
| **Conditions:** Posttraumatic stress disorder (PTSD), Anxiety and Depression. |  |  |  | **Neutral:** N/A. |
| **Study Citation:** (Kemp et al., 2022) | Assess the impact of a collaborative care intervention on anxiety symptoms among participants in India with comorbid depression, poorly controlled diabetes, and moderate to severe anxiety symptoms. | **Level of implication:** **Health system**: Diabetes clinics in urban India; **Provider:** Care coordinators, psychiatrists, diabetologists; **Patient:** Adults with depression, poorly controlled diabetes, and anxiety symptoms in India. | - **Health outcomes:** At 12 months, 65.7% of collaborative care patients vs. 41.4% of usual care patients had significant anxiety symptom reduction. Older patients had greater anxiety improvement at 24 months. - **Socio-behavioural:** No major differences in impact by sex, education, or income of the intervention. - **System (health system) level:** N/A. | **Positive:** Improved anxiety symptoms during intervention among patients with depression and diabetes. |
| **Country:** India. |  | **Aspects of CEI:**   - **Health Education:** Provided evidence-based behavioural activation and diabetes self-management counselling tailored to the Indian context. - **Community Participation:** Engaged patients through regular contacts, goal-setting, and involvement of families in care coordination. - **Collaborative Support**: Delivered integrated care via a multidisciplinary team including care coordinators, psychiatrists, and diabetologists working together. |  | **Negative:** No sustained anxiety symptom improvement post-intervention. |
| **Conditions:** Diabetes, Depression, and Anxiety. |  |  |  | **Neutral:** N/A. |
| **Study Citation:** (Halliday et al., 2024) | 1. Analyse self-stigma scores longitudinally to compare participants assigned to CoCM *vs.* enhanced standard-of-care.  2. Evaluate whether change in self-stigma over 12 months predicts type 2 diabetes outcomes at 12 months, and whether this relationship is mediated by depressive symptom severity. | **Level of implication:** **Health system:** Urban diabetes clinics in India; **Provider:** Integrated care teams including diabetes specialists, therapists, and care coordinators; **Patient**: Adults with depression and type 2 diabetes in urban India. | - **Health outcomes:** Change in self-stigma over 12 months predicted small but significant improvements in HbA1c (−0.07% per 1-point stigma decrease); depressive symptoms did not mediate this effect. - **Socio-behavioural:** Most participants (84%) identified diabetes, not depression, as the most stigmatizing condition; baseline self-stigma levels were low and changed minimally over time. - **System (health system) level:** CoCM implemented across 4 urban diabetes clinics with integrated behavioural health, did not significantly reduce self-stigma relative to enhanced standard care. | **Positive:** Showed improved depression and diabetes outcomes. |
| **Country:** India |  | **Aspects of CEI:**   - **Health Education:** Delivered integrated care with depression screening and culturally relevant self-management support for diabetes and depression. - **Community Participation:** Engaged patients through regular visits, goal-setting, and involvement of families in treatment. - **Collaborative Support:** Multidisciplinary teams including care coordinators, psychiatrists, and diabetologists to provide coordinated care. |  | **Negative:** Self-stigma did not significantly decrease or mediate diabetes outcomes. |
| **Conditions:** Diabetes and Depression |  |  |  | **Neutral:** N/A. |
| **Study Citation:** (Srinivasan, Salazar, et al., 2022) | Examine whether elevated concentration of total homocysteine (tHcy) at baseline predicts severity of depression at 6-month follow-up in patients diagnosed with depression and comorbid chronic medical conditions. | **Level of implication:** **Health system**: Rural PHCs; **Provider:** PHC staff trained in depression care; **Patient:** Adults with depression and chronic medical conditions in rural Karnataka. | - **Health outcomes:** Elevated homocysteine levels did not predict depression severity at 6 months; mean PHQ-9 scores were similar (7.4 *vs.* 7.6, p=0.67). - **Socio-behavioural:** N/A. - **System (health system) level:** Study conducted within enhanced standard care arm of a rural primary care trial integrating depression screening and management with chronic disease care. | **Positive:** N/A. |
| **Country:** India |  | **Aspects of CEI:**   - **Health Education:** Provided basic training to PHC staff for depression screening and management. - **Community Participation:** Included adults with depression and chronic conditions from rural communities with follow-up assessments. - **Collaborative Support:** Enhanced standard care compared to collaborative care by PHC teams aiming to integrate mental health treatment. |  | **Negative:** Elevated homocysteine not predictive of depression severity. |
| **Conditions:** Depression, Hypertension, Diabetes, or Ischemic heart disease (IHD) |  |  |  | **Neutral:** Study examined homocysteine levels and depression severity in rural primary care. |
| **Study Citation:** (Katon et al., 2010) | Determine whether coordinated care management of multiple conditions improves disease control. | **Level of implication:** **Health system:** Integrated health care system at primary care clinics; **Provider**: Nurses collaborating with primary care physicians; **Patient**: Adults with depression and poorly controlled diabetes and/or coronary heart disease. | - **Health outcomes:** At 12 months, intervention patients improved depression [Symptom Checklist (SCL)-20 −0.40], HbA1c (−0.58%), low-density lipoprotein (LDL) cholesterol (−6.9 mg/dL), and SBP (−5.1 mm Hg) significantly more than controls. - **Socio behavioural:** Intervention patients reported higher satisfaction with care; adherence to diet and exercise did not differ significantly. - **System (health system) level:** Nurse care managers worked with primary care physicians across 14 clinics delivering treat-to-target guideline-based care with frequent contacts. | **Positive:** Improved depression, HbA1c and SBP. |
| Countries: USA |  | **Aspects of CEI:**   - **Health Education:** Nurses provided guideline-based education and self-care materials to manage depression, diabetes, and heart disease. - **Community Participation:** Patients actively engaged through frequent nurse visits, goal-setting, and medication adherence coaching. - **Collaborative Support:** Multidisciplinary team including nurses, primary care physicians, and psychiatrists for coordinated integrated care. |  | **Negative**: N/A. |
| **Conditions:** Depression, Diabetes, Coronary Heart Disease |  |  |  | **Neutral:** N/A. |
| **Study Citation:** (Katon et al., 2004) | Determine whether enhancing quality of care for depression improves both depression and diabetes outcomes in patients with depression and diabetes. | **Level of implication:** **Health system:** Primary care clinics; **Provider:** Depression clinical specialist nurses; **Patient**: Adults with diabetes and comorbid major depression. | - **Health outcomes**: Collaborative care improved depression significantly but did not improve HbA1c. - **Socio-behavioural:** Patients had higher satisfaction and more adequate antidepressant treatment than usual care. - **System (health system) level:** Nurse care managers worked with primary care doctors using psychiatric supervision across the study setting. | **Positive:** Improved depression care and outcomes. |
| **Country:** USA. |  | **Aspects of CEI:**   - **Health Education:** Nurses provided guideline-based education and self-care materials to improve management of depression. - **Community Participation:** Patients actively engaged through frequent nurse visits, goal-setting, and medication adherence coaching. - **Collaborative Support:** A multidisciplinary team including nurses, primary care physicians, and psychiatrists coordinated integrated care for patients. |  | **Negative:** No improvement in HbA1c control. |
| **Conditions:** Diabetes and Depression. |  |  |  | **Neutral:** N/A. |
| **Study Citation:** (Parsons et al., 2000) | Describe the impact of a pharmacist–physician collaborative care model on patient outcomes and health services utilization. | **Level of implementation:** **Health system**: N/A; **Provider:** Counsellors and health educators; **Patient:** Men with haemophilia and HIV. | - **Health outcomes:** Theory-based intervention maintained safer sexual behaviours over time in haemophilia patients. - **Socio-behavioural:** Increased self-efficacy and perceived family support improved safer sex practices. - **System (health system) level:** Intervention was feasible and standardized across multiple haemophilia care centres. | **Positive:** Effective for maintaining safer sexual behaviours in haemophilia patients. |
| **Country:** USA |  | - **Health Education:** Participants received theory-based safer sex education focusing on behaviour maintenance strategies. - **Community Participation:** Participants engaged actively in group sessions and follow-ups reinforcing safer sexual practices. - **Collaborative Support**: Intervention was delivered by a multidisciplinary team coordinating education and support efforts. |  | **Negative:** N/A. |
| **Conditions:** HIV and Haemophilia |  |  |  | **Neutral:** N/A. |
| **Study Citation:** (Hegel et al., 2005) | To evaluate the effect of comorbid panic disorder and PTSD on response to a collaborative-care intervention for late-life depression in primary care. | **Level of implementation**: **Health system:** Primary care clinics; **Provider:** Nurse or psychologist depression care managers; **Patient:** Depressed older adults with co-morbid panic disorder or PTSD. | - **Health outcomes:** Collaborative care improved depression symptoms similarly in older adults with and without co-morbid panic disorder; improvement in those with PTSD was slower but significant by 12 months. - **Socio-behavioural**: Patients with co-morbid anxiety (panic or PTSD) had greater depressive severity, suicidal thoughts, functional impairment, and lower quality of life at baseline.   **System (health system) level:** Collaborative care model was effective and feasible for patients with and without co-morbid anxiety, though PTSD patients required longer, sustained intervention and proactive engagement. | **Positive:** Effective for late-life depression with co-morbid panic disorder and PTSD, although slower response in PTSD. |
| **Country:** USA |  | - **Health Education:** Depression care managers educated older adults about depression, treatment options, and behavioural activation. - **Community Participation**: Patients engaged in regular follow-ups, psychotherapy, and shared treatment preferences despite comorbid anxiety.   **Collaborative Support:** Care involved coordinated efforts among depression care managers, primary care physicians, and psychiatrists in a collaborative care model. |  | **Negative:** N/A. |
| **Conditions:** Depression, Panic Disorder, PTSD and Generalized Anxiety Disorder |  |  |  | **Neutral:** N/A. |
| **Study Citation:** (Matzke et al., 2018) | Describe impact of a pharmacist–physician collaborative care model on patient outcomes and health services utilization. | **Level of implementation:** **Health system:** Carilion Clinic hospitals and PCMH practices; **Provider:** Clinical pharmacists and physicians; **Patient:** Adults with multiple chronic conditions (≥2) on ≥4 medications. | - **Health outcomes:** Significant improvements in HbA1c, BP, and cholesterol levels; reduced hospitalizations by 23.4%. - **Socio-behavioural:** Patients empowered through pharmacist collaboration to self-manage medications and health conditions. - **System (health system) level:** Pharmacist–physician collaborative care model within PCMH reduced hospitalizations and yielded a 504% return on investment. | **Positive:** Improved clinical outcomes and reduced hospitalizations. |
| **Country:** USA |  | - **Health Education:** Clinical pharmacists provided medication management education for multiple chronic conditions. - **Community Participation:** Patients engaged through scheduled pharmacist visits and follow-ups for medication and health self-management.   **Collaborative Support:** Coordinated care between pharmacists, physicians, and care teams within PCMHs. |  | **Negative:** Non-significant increase in emergency department visits. |
| **Conditions:** Congestive Heart Failure (CHF), Hypertension, Hyperlipidemia, Diabetes, Asthma, COPD and Depression. |  |  |  | **Neutral:** N/A |
| **TFM: HBM (n = 4)** | | | | |
| **Study Citation:** (Wang et al., 2020) | Determine impact of a comprehensive reminder system intervention on health behaviours, medication adherence, BP, disability, and stroke recurrence in patients with hypertension who have had a stroke from baseline to 6 months after discharge. | **Level of implication:** **Health system:** Hospitals and follow-ups via phone; **Provider:** Nurses and doctors; **Patient:** Hypertensive stroke patients. | - **Health outcomes:** At 6 months, intervention improved health behaviours, medication adherence, SBP (−10.77 mmHg), and disability; stroke recurrence was low and similar to control group. - **Socio-behavioural**: Multi-method reminders boosted patient engagement and lifestyle habits. - **System (health system) level:** Nurses delivered ongoing education via calls, short message service (SMS), and printed materials across 3 hospitals, showing practical implementation. | **Positive:** Improved health behaviours, medication adherence, BP, and disability sustained till 6 months. |
| **Country:** China |  | **Aspects of CEI:**   - **Health Education:** Continuous multi-method education to improve health behaviours and medication adherence. - **Community Participation:** Active patient involvement through reminders, interviews, and self-management tools. - **Collaborative Support:** Nurses and healthcare providers coordinated ongoing support to improve outcomes. |  | **Negative:** No significant difference in stroke recurrence between groups. |
| **Conditions:** Hypertension and Stroke |  |  |  | **Neutral:** N/A. |
| **Study Citation:** (Wan et al., 2018) | Determine whether implementation of the comprehensive Reminder System (CRS)-HBM improves health behaviours and BP control among Chinese hypertensive stroke patients 3 months post hospital discharge from an occurrence of hypertensive ischemic stroke. | **Level of implementation:** **Health system:** Hospitals; **Provider:** Nurses and doctors; **Patient:** Hypertensive ischemic stroke patients. | - **Health outcomes:** Intervention improved physical activity, nutrition, low-salt diet, medication adherence, and lowered SBP significantly at 3 months; no effect on smoking or alcohol use. - **Socio-behavioural:** Multi-method reminders enhanced health beliefs, motivation, and engagement in behaviour change. - **System (health system) level:** Nurses delivered combined face-to-face, telephone education, and weekly automated SMS reminders in three hospitals; feasible and effective for mild stroke patients. | **Positive:** Improved health behaviours and BP control. |
| **Country:** China |  | **Aspects of CEI:**   - **Health Education:** Multi-method education improved health behaviours and medication adherence. - **Community Participation:** Patients actively engaged through reminders and follow-ups. - **Collaborative Support:** Nurses and providers coordinated ongoing care to enhance outcomes. |  | **Negative:** No significant changes in smoking or alcohol use behaviours. |
| **Conditions:** Hypertension and Stroke |  |  |  | **Neutral:** N/A. |
| **Study Citation:** (Ansari et al., 2020) | Activate patients with COPD in relation to their health behaviour by delivering a tailored, self-management support program that acknowledges and addresses the implications of co-morbidities, within the primary care setting. | **Level of implementation:** **Health system:** N/A; **Provider**: General practice nurses (PNs); **Patient**: COPD patients with multi-morbidity. | - **Health outcomes**: Improved COPD-related quality of life (QoL), enhanced knowledge. - **Socio-behavioural**: Increased patient activation and self-efficacy for health behaviour. - **System (health system) level:** N/A. | **Positive**: Improved patient activation, COPD knowledge, quality of life, and inhaler technique. |
| **Country:** Australia |  | - **Health Education**: Practice nurses delivered tailored self-management education to patients with COPD and multi-morbidity. - **Community Participation**: Patients were actively engaged in face-to-face sessions, discussing individual health priorities and self-management. - **Collaborative Support**: Intervention involved coordination between general practitioners, practice nurses, and patients within primary care practices. |  | **Negative:** No significant change in patients' perception of multimorbidity or COPD-related multimorbidity. |
| **Conditions:** Chronic Obstructive Pulmonary Disease (COPD), and multi-morbidity. |  |  |  | **Neutral:** Single-group study without control group limits attribution of effects solely to the intervention. |
| **Study Citation:** (Hing et al., 2019) | Identify and explore personal, interpersonal, and structural factors influencing adherence to both antiretroviral therapy (ART) and antihypertensive medication among people living with HIV and hypertension in Malawi, using the HBM. | **Level of implementation:** **Health system:** Health clinic; **Provider:** Doctors and counsellors; **Patient:** Adults with HIV and hypertension. | - **Health outcomes:** Poorer medication adherence and uncontrolled BP for hypertension compared to HIV, despite recognition of hypertension severity. - **Socio-behavioural:** Patients perceive hypertension as equally or more severe than HIV, influenced by personal and community illness experiences; financial burden and lifestyle change challenges affect hypertension adherence. - **System (health system) level:** Free ART but costly hypertension medications create structural barriers to adherence; lack of integrated care and medication affordability issues. | **Positive:** HBM applied to HIV and hypertension adherence behaviours. |
| **Country:** Malawi |  | - **Health Education:** Participants received counselling on hypertension and HIV treatment, including lifestyle changes and medication adherence. - **Community Participation:** Patients shared experiences and perceptions about their diseases within their social circles, influencing acceptance and management. - **Collaborative Support:** Integration of HIV and hypertension care at the clinic involved healthcare providers coordinating services for co-morbid patients. |  | **Negative:** N/A. |
| **Conditions:** HIV and Hypertension |  |  |  | **Neutral:** N/A. |
| **TFM: Improving Mood–Promoting Access to Care (IMPACT), (n = 1)** | | | | |
| **Study Citation:** (Harpole et al., 2005) | Determine whether or not the presence of multiple co-morbid medical illnesses affect patient response to a multidisciplinary depression treatment program. | **Level of implementation:** **Health system:** Primary care clinics; **Provider:** Depression clinical specialists (nurses/psychologists); **Patient:** Depressed older adults (≥60 years) with multiple chronic illnesses. | - **Health outcomes:** Care improved depression severity, remission rates, mental health scores, and QoL in older adults regardless of multiple chronic medical co-morbidities. - **Socio-behavioural:** Patients with more co-morbidities responded well to intervention without needing more intensive support. - **System (health system) level:** Multidisciplinary depression management integrated into primary care was feasible and effective across diverse health care settings. | **Positive:** Reducing depression severity and improving quality of life in older adults with multiple comorbid medical illnesses. |
| **Country:** USA |  | - **Health Education:** Depression care managers provided education about depression, treatment options, and behavioural activation tailored for older adults. - **Community Participation**: Patients actively engaged through regular follow-ups, psychotherapy sessions, and shared treatment preferences. - **Collaborative Support:** Coordinated care involving depression care specialists, primary care physicians, psychiatrists, and other healthcare providers central to the intervention delivery. |  | **Negative**: N/A. |
| **Conditions:** Depression and associated comorbidities |  |  |  | **Neutral:** Presence of multiple chronic illnesses did not affect response to depression treatment; study based on self-reported co-morbidity. |
| **TFM: Transtheoretical Model (TTM), (n = 2)** | | | | |
| **Study Citation:** (Butler et al., 2003) | Develop and evaluate theory-based interventions designed to change sexual behaviour and promote safer sex practices of HIV seropositive young men and adolescents with haemophilia to prevent transmission to sexual partners and offspring. | **Level of implementation: Health system:** Haemophilia care sites; **Provider:** Psychologists, social workers, health educators, nurses; **Patient:** HIV-positive adolescents and young men with haemophilia. | - **Health outcomes**: Increased safer sex behaviour with 79% in action or maintenance stage post-intervention; sustained over 6 months. - **Socio-behavioural:** Improved self-efficacy for condom use, outercourse, and abstinence; greater perceived family support for safer sex. - **System (health system) level:** Standardized, theory-based interventions feasible across multiple haemophilia care sites without a control group. | **Positive:** Effective stage-based, theory-driven safer sex behaviour change for HIV-positive youth with haemophilia. |
| **Country:** USA |  | - **Health Education:** Individual and group sessions provided stage-based safer sex education using the TTM and Theory of Reasoned Action. - **Community Participation:** Participants engaged in peer group discussions, social activities, and weekend retreats to build supportive networks. - **Collaborative Support:** Multi-disciplinary teams of psychologists, nurses, and social workers coordinated intervention delivery across multiple care sites. |  | **Negative:** N/A. |
| **Conditions:** HIV and Haemophilia |  |  |  | **Neutral**: N/A. |
| **Study Citation:** (Velasquez et al., 2009) | Evaluate the efficacy of a theory-based intervention designed to reduce both alcohol use and incidence of unprotected sexual behaviours among HIV-positive MSM with alcohol use disorders. | **Level of implementation**: Health system: N/A; **Provider:** Therapists and peer counsellors; **Patient:** HIV-positive MSM with alcohol use disorders. | - **Health outcomes:** Intervention reduced number of drinks, heavy drinking days, and days of combined heavy drinking and unprotected sex over 12 months. - **Socio-behavioural:** Increased motivation and confidence to change alcohol use and safer sex behaviours through individualized and group counselling. - **System (health system) level:** Integrated manualized intervention combining motivational interviewing and peer support feasible and effective in HIV care settings. | **Positive:** Helped reduce alcohol use and unprotected sex. |
| **Country:** USA |  | - **Health Education:** Individual counselling and peer group sessions taught alcohol reduction and safer sex using the Transtheoretical Model and motivational interviewing. - **Community Participation:** Participants engaged in peer-led group discussions and activities to support behaviour change. - **Collaborative Support:** Intervention involved coordinated efforts between therapists, trained peer counsellors, and participants. |  | **Negative:** N/A. |
| **Conditions:** HIV and Alcohol use disorder |  |  |  | **Neutral:** N/A. |
| **TFM: Patient-Centered Medical Home (PCMH), (n = 1)** | | | | |
| **Study Citation:** (John et al., 2020) | Evaluate changes in the mean patient activation scores and investigate significant predictors of patient activation following a 12-month PCMH intervention among individuals presenting with one or more chronic diseases. | **Level of implementation: Health system:** Six general practices in Sydney; **Provider:** General practitioner (GPs) and chronic disease management care coordinators; **Patient**: Adults with one or more chronic diseases. | - **Health outcomes:** Significant improvement in patient activation scores after 12 months in chronic disease patients. - **Socio-behavioural:** Older age and lack of private insurance associated with lower activation; increased baseline activation predicted better follow-up activation. - **System (health system) level:** GP-led multidisciplinary team care with tailored chronic disease management was feasible and effective in Australian primary care. | **Positive:** Improved patient activation and self-management in chronic disease patients. |
| **Country:** Australia |  | - **Health Education:** Individualized self-management education delivered by GPs and trained care coordinators. - **Community Participation**: Patients actively engaged in co-designing care plans and using digital tools for ongoing support. - **Collaborative Support:** Coordinated team-based care involving GPs, chronic disease management coordinators, and digital health platforms. |  | **Negative:** No control group to confirm causality; follow-up Patient Activation Measure (PAM) scores still in moderate activation range. |
| **Conditions:** Chronic diseases in general |  |  |  | **Neutral**: N/A. |
| **TFM: Patient-Centered Care Model (PCCM), (n = 1)** | | | | |
| **Study Citation:** (Hickey et al., 2021) | Evaluate the effect of the Sustainable East Africa Research in Community Health (SEARCH) patient-centered, streamlined care intervention on mortality and hypertension care cascade outcomes among adults with uncontrolled hypertension identified at baseline population-level. | **Level of implementation: Health system:** Government-run health facilities with integrated chronic care; **Provider:** Nurses, clinical officers, and trained clinic staff; **Patient:** Adults with uncontrolled hypertension in rural Kenya and Uganda. | - **Health outcomes:** Patient-centered integrated hypertension and HIV care reduced 3-year all-cause mortality by 21% and improved hypertension control by 22%. - **Socio-behavioural:** Community-based multi-disease screening increased hypertension diagnosis and linkage to care, with higher engagement among those with severe hypertension and HIV. - **System (health system) level:** Integration of hypertension care into existing HIV platforms with streamlined, patient-centered services feasible and effective in rural Kenyan and Ugandan health systems. | **Positive:** Reduced 3-year all-cause mortality by 21% and improved hypertension control by 22%. |
| **Country:** Kenya and Uganda |  | - **Health Education:** Provided patient-centered, culturally tailored hypertension education integrated with HIV care. - **Community Participation:** Participants engaged through community-wide hypertension screening and linkage support. - **Collaborative Support:** Coordinated care with clinic staff, nurses, and the HIV care system delivering integrated chronic disease management. |  | **Negative:** Half of participants still had uncontrolled hypertension despite intervention. |
| **Conditions:** Hypertension and Diabetes |  |  |  | **Neutral:** N/A. |
